# Corona Concerts: The Effect of Virtual Concert Characteristics on Social Connection and *Kama Muta*

**DOI:** 10.3389/fpsyg.2021.648448

**Published:** 2021-06-22

**Authors:** Dana Swarbrick, Beate Seibt, Noemi Grinspun, Jonna K. Vuoskoski

**Affiliations:** ^1^RITMO Centre for Interdisciplinary Studies in Rhythm, Time and Motion, University of Oslo, Oslo, Norway; ^2^Department of Musicology, University of Oslo, Oslo, Norway; ^3^Department of Psychology, University of Oslo, Oslo, Norway; ^4^Centro de Investigação e Intervenção Social (CIS-IUL), Instituto Universitário de Lisboa (ISCTE-IUL), Lisbon, Portugal; ^5^Departamento de Música, Universidad Metropolitana de Ciencias de la Educación, Santiago, Chile

**Keywords:** coronavirus, COVID-19, live, music, concerts, virtual, social connection, *kama muta*

## Abstract

The popularity of virtual concerts increased as a result of the social distancing requirements of the coronavirus pandemic. We aimed to examine how the characteristics of virtual concerts and the characteristics of the participants influenced their experiences of social connection and *kama muta* (often labeled “being moved”). We hypothesized that concert liveness and the salience of the coronavirus would influence social connection and *kama muta*. We collected survey responses on a variety of concert and personal characteristics from 307 participants from 13 countries across 4 continents. We operationalized social connection as a combination of feelings and behaviors and *kama muta* was measured using the short *kama muta* scale (Zickfeld et al., 2019). We found that (1) social connection and *kama muta* were related and predicted by empathic concern, (2) live concerts produced more social connection, but not *kama muta*, than pre-recorded concerts, and (3) the salience of the coronavirus during concerts predicted *kama muta* and this effect was completely mediated by social connection. Exploratory analyses also examined the influence of social and physical presence, motivations for concert attendance, and predictors of donations. This research contributes to the understanding of how people can connect socially and emotionally in virtual environments.

## Introduction

“In today’s world of fear and unease and social distancing […] I don’t know when it will be safe to return to singing arm in arm at the top of our lungs, hearts racing, bodies moving, souls bursting with life. But I do know that we will do it again, because we have to […] We need moments that reassure us that we are not alone” (Grohl, 2020).

Musicians responded rapidly to governments’ social distancing restrictions during the coronavirus pandemic by providing streamed concerts. These streamed concerts seemed to provide a sense of comfort and connection during the uncertain and challenging times. To the best of our knowledge, the elements that facilitate the sense of connection and closeness remain unexplored. We aimed to examine a variety of concert characteristics and personal traits to understand what elements of virtual concerts facilitate social connection in concert viewers.

Virtual concerts typically consist of one or more musicians performing to a video camera and audiences watch on their personal devices, such as laptops or smartphones. The video of the performance may be livestreamed to a viewing platform such as a social media website or it could be posted after recording as a pre-recorded performance. Several platforms exist for artists to stream their virtual concerts including YouTube, Facebook, Instagram, Twitch, and Zoom. Concerts on YouTube, Facebook, and Instagram could be livestreamed or pre-recorded while Twitch and Zoom typically offer only livestreamed content. The social media platforms often contain features that allow audience members to interact with each other and performers in ways that are distinct from the traditional live concert such as with a chat feature or reaction buttons. Many arts organizations also began to host livestreamed or pre-recorded concerts on their own websites to connect with audiences. Additionally, there were some new companies that formed during the lockdown to provide artists with additional platforms to monetize their performances (Breese, 2020).

The distinction between live and pre-recorded performances is important because research on live concert experiences at real-world concert venues suggests that audiences engage differently with live and pre-recorded performances. Live performances offer a shared and novel experience for audiences, which is something so desirable that cost is not a factor when choosing to attend concerts (Brown and Knox, 2017). Live audiences move more vigorously, have stronger musical experiences, and have the opportunity to interact directly with the performer, possibly strengthening parasocial relationships between the audience and performers (Lamont, 2011; Pehkonen, 2017; Baym, 2018; Kurtin et al., 2019; Swarbrick et al., 2019). Understanding the effects of virtual concerts is important because research suggests that live musical events are beneficial to health and well-being (Fancourt and Williamon, 2016; Fancourt and Steptoe, 2018).

The distinction between live and pre-recorded music may be less clear in the virtual concert environment because streaming platforms such as YouTube and Facebook have introduced features such as the “Live chat replay” where even after a concert has been livestreamed, viewers can see comments appear as they would have in the livestreamed concert. Features like this may create feelings of being in the presence of others even while watching alone. Therefore, we collected information not only on whether a concert was livestreamed or pre-recorded, but also on whether it was a live-streamed concert being viewed in real time or after the concert was aired.

Certain genres may be better suited to the livestream context than others. Specifically, it may be easier for musicians in certain genres to create a virtual auditory experience that is more similar to a real live concert than it would be for others due to the constraints of requiring the appropriate sound equipment. For example, electronic dance music producers who rely only on their computers may have been able to master the virtual stage faster than musicians in other genres.

Audio quality and video quality vary widely depending on the resources that musicians have available. Some performers could only use their smartphones to record or stream while others had access to sophisticated audio systems to deliver a higher quality experience. Some performers may have already had audio recording and streaming experience, however, others may have found themselves scrambling to make up for canceled gigs by quickly trying to master these technologies. Research suggests that audio quality is important for musical enjoyment, especially when listening to new music (Schoeffler et al., 2013), however, audio quality may have little effect on music video appreciation (Schoeffler and Herre, 2014). Nonetheless, audio quality may be an important predictor of presence or feelings of “being there” (Lessiter and Freeman, 2001; Nordahl and Nilsson, 2014). Presence has been categorized into subdomains such as physical and social presence (Makransky et al., 2017). Previous research suggests that feelings of social presence are important for feeling social connectedness (Rettie, 2003). Therefore, presence may be important for the virtual concert experience as it is in other communication media (IJsselsteijn et al., 2007).

The performers’ struggles making up for canceled gigs were shared by audience members who were being encouraged or forced to stay at home and to socially distance. People across the world lost their jobs and their loved ones and this suffering has been shared beyond borders. The severity of the coronavirus differed across countries depending on the regulations created by governments (Hale et al., 2020). Performers and audiences may have discussed their shared experiences during virtual concerts or encouraged donations to charities or for themselves. Research suggests that reminders of threats strengthen groups (Jackson et al., 2019; Gelfand et al., 2020). Therefore, we predicted that audience members’ perceptions of the salience of the coronavirus during the concert (for example if they noticed performers or audience members discuss the topic or encourage donations) would increase their sense of social connection during a virtual concert.

We additionally aimed to explore how the impact of the pandemic on individual participants (how frequently they felt loneliness, lack of companionship, isolated from others, and anxiety since the beginning of the social distancing measures) affected their responses to the virtual concerts. Maintaining social connection with others is important for both mental and physical wellbeing, and even moderate levels of social isolation can increase the incidence of clinical depression, suicidal thoughts, and stress (e.g., Adam et al., 2006; Heinrich and Gullone, 2006). By reducing social connectedness, the social distancing measures implemented to combat the global COVID-19 pandemic may thus have had detrimental effects on both mental and physical health. Indeed, emerging research suggests that the increased loneliness associated with COVID-19 lockdown measures has contributed to increased levels of depression and anxiety even in those with no pre-existing psychopathologies (Hoffart et al., 2020).

In the absence of face-to-face social contact, people may try to alleviate loneliness by engaging with mass or social media (Rubin and Rubin, 1985; Pittman and Reich, 2016; Sirola et al., 2019) or by listening to music (Taruffi and Koelsch, 2014; Schäfer et al., 2020). Even solitary music listening can be a social experience, conveying a sense of presence of another person. This sense of social connection can take the form of identifying or empathizing with the performer or composer (Scherer and Zentner, 2001), nostalgic reminiscence involving close social relationships (Garrido and Davidson, 2019), or experiencing the music itself as a virtual person (Levinson, 2006; Van den Tol and Edwards, 2013). Examples of the latter include experiencing music as a surrogate for an empathic friend, providing solace and understanding when one is working through negative emotions (Lee et al., 2013; Van den Tol and Edwards, 2013), and reducing experienced loneliness (Schäfer et al., 2020).

With the proliferation of technologies enabling live-streaming and virtual concert experiences, the social dimensions of ‘solitary’ music listening have expanded even further such that individuals can watch concerts while physically alone, but virtually together (cf. Charron, 2017). While attending real-life concerts is often socially motivated (e.g., Wilks, 2011), and live concert attendance can create and foster social bonds through shared emotional experiences and synchronous movement such as dancing (e.g., Tarr et al., 2016; Savage et al., 2020), it is not yet known whether such effects can also be obtained in online concert environments. However, research focusing on online social networking platforms has shown that social media use can yield many of the same benefits (such as felt social connection and perceived social support) as face-to-face social interaction, as long as the usage itself is active, meaningful, and connection-promoting (Clark et al., 2018). In virtual concerts, viewers can actively engage and connect with others by “liking” the video, commenting, and sharing with their friends/followers (Verduyn et al., 2015).

In daily life, we may feel moved by works of art or by events or actions that we witness. Recent empirical work has established that feelings of being moved are primarily experienced as positive, are typically elicited by significant social or moral events and works of art, and motivate social bonding and devotion to communal relations (see e.g., Menninghaus et al., 2015; Zickfeld et al., 2019). *Kama muta* (Sanskrit term for ‘moved by love’) is a construct that conceptualizes the warm, positive emotion that we often label as *being moved* as a social relational emotion, which also encompasses a range of other, related labels such as *heartwarming, nostalgia*, and *love* (Zickfeld et al., 2017). *Kama muta* is thought to occur when communal sharing relationships (CSRs) suddenly intensify (Fiske et al., 2019). CSRs are relationships in which individuals feel equal and they share according to need and ability. CSRs can be communicated through closeness, touch, and synchrony, for example. *Kama muta* can be evoked by experiences such as being reunited with a close friend, remembering a loved one at a funeral, seeing others experience *kama muta*, and watching videos of cute vulnerable animals (Fiske et al., 2019). It is hypothesized that several cultural practices, such as sharing ritual food and drink during Eucharist or the kiss of bride and groom during a wedding ceremony, exist to evoke *kama muta* and subsequently strengthen CSRs (Fiske et al., 2019).

*Kama muta* can also be felt at a valuable experience of social connection against a backdrop of loss or temporary lack of important relationships (Fiske et al., 2019). It is possible, through this mechanism, that when performers discuss the coronavirus during concerts, participants may experience *kama muta* when the music and musician provides them with a comforting feeling of connectedness in sharp contrast to the negative reminder of the global pandemic. In line with this, participants were more moved by vignettes describing people getting close to each other in unfavorable circumstances than in favorable ones (Strick and van Soolingen, 2018). Additionally, during the pandemic, performers may have stated that they felt togetherness with the audience through their shared struggles, which could result in *kama muta* through emotional contagion (Hatfield et al., 1994). A collective awareness and discussion of the pandemic may thus evoke *kama muta* through the solidarity evoked by a common fate, through the comfort of sharing a beautiful experience amidst a crisis, and through emotional contagion of others’ *kama muta*. Accordingly, we predicted that the more the pandemic was made salient during a concert the more connected people would feel, and this in turn would increase their *kama muta* responses. Thus, we hypothesized that connectedness would mediate the effect of coronavirus salience on *kama muta*.

Music is frequently reported to evoke feelings of being moved. In a questionnaire study investigating listeners’ typical responses to music, feeling moved was found to be the fourth most common emotional response to music listening (Juslin and Laukka, 2004). Although it might not be immediately apparent how music listening could evoke experiences of intensified communal sharing, there are multiple ways in which engagement with music can evoke *kama muta:* A listener might identify with or feel a connection to the music in general, for example through empathy or rhythmic entrainment (e.g., Trost et al., 2017; Huron and Vuoskoski, 2020), as well as to the composer, the musicians, or other fans/listeners. Listening to familiar music might also evoke nostalgic feelings by reminding us of important social relationships. To date, empirical research on music-evoked feelings of being moved has focused mainly on investigating solitary listening experiences. This research has established that feelings of being moved are important in mediating the enjoyment of sad-sounding music (Vuoskoski and Eerola, 2017). These findings mirror those obtained in other domains; feelings of being moved have also been shown to mediate the enjoyment of tragic films (Hanich et al., 2014). Furthermore, emerging research suggests that music-evoked experiences of feeling moved are associated with a similar pattern of appraisals (such as experiencing a sense of connection) and physiological sensations as feeling moved by videos depicting social scenarios (Vuoskoski et al., 2012; Schubert et al., 2018).

Empathy may be an important predictor for participants’ tendency to feel social connection and *kama muta* in concerts. Empathic Concern is one of the four subscales of the *Interpersonal Reactivity Index* (with the other three being Fantasy, Perspective-taking, and Personal Distress; Davis, 1980), a widely used measure of trait empathy. Empathic concern taps into the tendency to experience feelings of sympathy and compassion for others undergoing negative experiences (Davis, 1980), and represents the facet of empathy that is most reliably associated with prosocial behavior and altruistic helping (e.g., FeldmanHall et al., 2015). Since empathic concern also predicts experiences of feeling moved, Zickfeld et al. (2017) suggested that empathic concern may moderate experiences of *kama muta* by facilitating identification with others in need, or that the intentions to act prosocially – evoked by empathic concern – might in themselves represent sudden intensifications of communal sharing (thus eliciting *kama muta*). In this sense, feelings of empathic concern could be considered a part of the broader *kama muta* construct (Zickfeld et al., 2017).

In the domain of music, empathic concern has been associated with enjoyment of sad and tender music, as well as with the intensity of emotions evoked by sad music (Vuoskoski et al., 2012; Eerola et al., 2016; Vuoskoski and Eerola, 2017). Further research has revealed that empathic concern appears to contribute to the enjoyment of sad music chiefly by intensifying feelings of being moved (Vuoskoski and Eerola, 2017). Interestingly, recent research has also associated empathic concern with the amount of spontaneous body movement elicited by electronic dance music (Zelechowska et al., 2020). This suggests that empathic concern may also be related to enhanced motor resonance in the context of music listening.

In sum there are a number of concert and personal variables that may influence attendants’ experiences of social connection and *kama muta*. To address the question of what aspects of the virtual concert contribute to experiences of social connection and *kama muta*, we collected survey responses from participants who had recently watched at least 15 minutes of a virtual concert. We formulated three main hypotheses and conducted additional exploratory analyses. Specifically, we hypothesized that (1) social connection and *kama muta* are related, and are predicted by empathic concern (see Zickfeld et al., 2017), (2) livestreamed concerts would lead to more social connection and *kama muta* than pre-recorded concerts, and (3) greater salience of the coronavirus pandemic would facilitate social connection which would mediate the effect on *kama muta*.

## Materials and Methods

Participants were recruited to participate in a research project titled “Quarantine Concerts” though participants were not necessarily in quarantine. The participants were invited to take a survey if they had recently watched at least 15 minutes of a virtual concert and they were encouraged to watch a concert of their choice if they had not recently done so. The concerts that participants watched could have occurred before the coronavirus pandemic or they could have occurred during the pandemic; however, all concert viewing took place during the worldwide coronavirus pandemic. Participants were encouraged to think about their last online concert experience when they responded to the questions and thus responses were retrospective. This study was approved by the ethics committee of the University of Oslo’s Department of Psychology. All participants gave written informed consent in accordance with the Declaration of Helsinki, 2013.

### Participants

Participants were recruited with a variety of methods through targeted social media advertising, mailing lists, and industry partnerships. Social media advertisements were targeted to individuals with an interest in music who were located in Norway, Germany, Chile, Canada, the United States, India, United Kingdom, and Spain. Recruitment occurred between May 3rd and July 15th, 2020, but the dates of targeted advertising varied across countries. Exclusion criteria were being a minor (under 18 years old) and having watched less than 15 minutes of a virtual concert. Participants who completed the survey could participate in a lottery to win one of twenty gift cards of value 200 NOK, 20 EUR, 20 USD, 25 CAD, 15000 CLP, 15 GBP, or 1500 INR depending on the individual’s country. Industry organizations and musicians were invited to participate as partners by promoting the study through their concerts. In exchange, they were promoted on the study’s webpage^1^ and in the InSync Lab’s social media (@InSyncLab).

661 people clicked on the link to participate in the research and provided consent to participate. In the survey, participants were first asked if they had recently watched a concert for more than 15 minutes. If they responded “No” they were advised that it is important that they have adequate experience with online concerts and to spend at least 15 minutes watching an online concert of their choice before answering the survey. After excluding for not having watched >15 minutes of a concert (*n* = 124 who responded “No” to the first question, *n* = 7 who filled the survey but reported spending less than 15 min watching a concert), being a minor (*n* = 15), providing nonsense responses (*n* = 3), or not completing the social connection and *kama muta* items (*n* = 205), the final sample size was *n* = 307 (Woman: *n* = 156, Man: *n* = 147, Prefer not to say: *n* = 3, Agender: *n* = 1). Participants resided in Chile (*n* = 185), Norway (*n* = 59), Canada (*n* = 20), United States (*n* = 9), India (*n* = 9), Germany (*n* = 8), United Kingdom (*n* = 6), Spain (*n* = 5), the Netherlands (*n* = 2), Denmark (*n* = 1), Hungary (*n* = 1), Malaysia (*n* = 1), and Switzerland (*n* = 1). In the final sample, *n* = 76 had watched a pre-recorded concert, *n* = 158 a live-streamed concert when it was aired and *n* = 65 a live-streamed concert after it had been aired, forming the three levels of our main variable concert type.

### Questionnaire Design

The questionnaire consisted of questions on participants’ demographics, the personal impact of the coronavirus, their musical background, the concert characteristics, their motivations for concert attendance, and the outcome measures of social connection and *kama muta*. Questions were answered on 5-point Likert scales (1–5), unless otherwise noted. The survey was translated from English into Norwegian, German, and Spanish (see Supplementary 1). Participants reported demographics of age, gender, and country of residence. They also reported how frequently they attended real, in-person concerts before the pandemic and how frequently they had watched virtual concerts that were video recorded and live-streamed in the past month. Concert characteristics included the viewing platform as text input (e.g., Facebook, YouTube), the duration participants watched (minutes), musical genre (text input), and the country of the concert. Participants selected or described the audio and video equipment they used and they reported their perceived quality of the audio and video. They reported their level of attention during the concert (i.e., background only vs. total concentration). Participants estimated the size of the audience and reported whether they knew anyone else in the audience and the number of other people watching in the same physical space (text). Participants also reported on the personal impact of the performer and their music by providing the extent to which they were a fan of the artist and the importance of the artists’ music. They also reported on the performer(s)’ level of interaction with each other and the audience. Participants shared how the concert made them feel with a free text response and by responding to feeling scales (e.g., peaceful, energetic, happy, sad, tense, and tender) (Vuoskoski and Eerola, 2017) and they reported on the extent to which they experienced embodied reactions of desiring to move along to the music, laughing out loud, and relaxed breathing. Participants reported on their appraisal of the concert by reporting whether the experience in the virtual concert seemed similar to their experiences in a real concert and whether they thought the concert was good (not at all vs. extremely; labeled concert quality hereafter).

The survey aimed to measure the personal impact of the coronavirus by asking participants how frequently they experienced loneliness, lack of companionship, isolation from others, and anxiety since the start of the pandemic (adapted from Hughes et al., 2004; Yang and Victor, 2011). *Kama muta* was measured with the KAMMUS-S which is the short version of the KAMMUS-2 (adapted from Zickfeld et al., 2019). The KAMMUS-S was translated from English into Spanish, however, questions from the German and Norwegian KAMMUS-2 were used to deliver the KAMMUS-S in German and Norwegian (Zickfeld et al., 2019). The survey also measured motivations for concert viewing (adapted from Rubin, 1983; Krause et al., 2014), the Multidimensional Presence Scale (Makransky et al., 2017), the Empathic Concern subscale from the Interpersonal Reactivity Index which measures trait empathy (Davis, 1980), and several items from the Musical Preference Questionnaire related to listening habits (Litle and Zuckerman, 1986). Specifically, participants’ attention levels during the concert, usual attention level while listening to music, number of hours listening to music per day, and the personal importance of music in participants lives during the past 3 years were measured with items that were adapted from the Musical Preference Questionnaire (Litle and Zuckerman, 1986). Participants reported whether they practice music and the number of months of musical training they have received.

One important concert characteristic was the salience of the coronavirus crisis. This could vary because participants could have watched concert videos filmed before or during the coronavirus pandemic, because the pandemic and the infection control measures had milder and more severe phases in the different regions examined, and because the hosts, performers, or audiences could engage with the topic to various extents. We measured coronavirus salience with a single item that asked: “How salient were the coronavirus-induced social distancing circumstances (e.g., did the performer or audience members discuss the topic or encourage donations)? 1 (Not at all salient) – 5 (Very Salient)”. Translations of this question used words that more closely approximated “prominent” which is a synonym of salient.

The short *kama muta* scale (KAMMUS-S) contains 11 items that reflect the 5 aspects of *kama muta*: (i) valence (*n* = 1; e.g., “I had positive feelings”), (ii) physical sensations (*n* = 5; e.g., chills or shivers), (iii) motivations or action tendencies (*n* = 2; e.g., “I wanted to hug someone”), (iv) appraisal patterns (*n* = 2; e.g., “I observed an exceptional sense of closeness appear”), (v) labels (*n* = 1; i.e., words used to describe *kama muta*) (Zickfeld et al., 2019). Due to an error with survey creation, instead of the *kama muta* scale having a response scale from 0 (Not at all) to 6 (A lot) as per its original design, it had response options of 1 (Not at all) to 6 (A lot).

We created a measure of social connection consisting of 3 aspects: (i) feelings, (ii) social presence, and (iii) behaviors measured by 10 items. In the dimension of feelings, we included questions on how connected participants felt to the performers and audience and whether they shared emotions with the audience. In the dimension of social presence, we chose two items from the social presence subscale of the Multimodal Presence scale to assess how much participants felt like they were in the presence of others and how much they felt others were aware of their presence (Makransky et al., 2017). The behaviors dimension contained items that gathered how much participants engaged in interactions surrounding the concert by reading, commenting, sharing, or discussing.

### Analysis

#### Pre-processing

Responses were pre-processed and analyzed in R (v4.0.2, 2020) and RStudio (v1.0.153, 2016). Free text responses were translated into English with assistance from native speakers (Spanish, Norwegian) and with assistance from Google Translate (German). Details on pre-processing can be found in Supplementary 2.

Alpha was used as a measure of reliability for several scales. Reliability was reasonable across the 10 social connection items (alpha = 0.81) and the 11 *kama muta* scale items (alpha = 0.87), therefore the values were averaged to create separate measures of social connection and *kama muta*. Measures reflecting potential effects of the pandemic on mental health (loneliness, lack of companionship, isolation from others, and anxiety) demonstrated reasonable reliability as well (alpha = 0.84), therefore they were averaged and are referred to in the results as “loneliness.”

The social media platform was determined from the URL or participant reports. Participants’ free text responses reporting the concert setting were re-coded to the categories of home, concert hall, and outdoors. Similarly, participants’ reported genres were re-coded into the most popular categories present in participants’ responses: African-American (consists of Soul/RandB/Funk, Jazz, Rap/hip-hop), Classical, Dance/electronica, Folk, Metal, Opera, Pop, Rock, and Various. Participants’ estimates of the size of the audience were manually re-coded to provide a numeric estimate of audience size. A principal component analysis revealed that re-wording the multimodal presence scale to make it relevant to the virtual concert environment caused a different loading structure such that one item from the social presence subscale was more relevant for the physical presence dimension. If participants watched a concert that occurred in the same country where participants were residing, this was identified as a shared country between the performer and the participant. Country of the participant was also re-coded into a variable “region” that classified whether participants were from Asia, Europe, North America, or Chile.

Loneliness, lack of companionship, isolation, and anxiety scores were averaged into an aggregate loneliness measure. To create the social connection variable, some variables needed to be adjusted before being averaged. Specifically, to measure commenting behavior, participants were asked “To what extent did you engage in interactions surrounding the concert by reading or commenting?”. Participants could select multiple response options explaining their commenting behavior from this list: 1 (I did not comment at all), 2 [I clicked on a reaction button (e.g., like)], 3 (I wrote a brief comment), 4 (I wrote multiple brief comments), 5 (I wrote one or more detailed comments). Participants could also select multiple response options explaining their sharing behavior from this list: 1 (I did not share the concert online), 2 (I shared the concert on a social media platform), 3 (I shared it directly with family/friends). We believe that each behavior is increasingly social, therefore we simply extracted the highest response option for these questions. The three sharing behavior response options were normalized to a 5-point scale (see the open science foundation repository for the pre-processing script^2^ and Supplementary 2 for the pre-processing details).

Responses to the question “How much of the concert did you watch? (minutes)” were manually converted to number of minutes if participants responded with words or in the unit of hours. If participants wrote that they had watched the full concert and they provided a link, the duration of watching was extracted from the length of the video. If people did not provide the link to the concert video or if the concert video was not available, these responses were not considered when examining concert viewing duration.

With regards to the question “How many months of musical training have you received?”, participants sometimes responded with words or in the unit of years therefore these were examined and re-coded manually when months could be extracted from participants’ responses. If the number of months of musical training could not be extracted from participants’ responses (e.g., “many years”) then these responses were not considered when examining the effect of musical training.

To assess the effectiveness of the industry and artist collaborations, partners’ names were searched in the responses. These references may appear in the URL in the case of the industry partnerships and in the name of the artist for the artist partnerships.

#### Statistical Analysis

Statistical analyses were performed in R with assistance from the toolboxes psych (Revelle, 2020) and tidyverse (Wickham et al., 2019). To examine the connections between empathic concern, social connection, and *kama muta*, we used Pearson correlations and simple linear regressions. Separate one-way ANOVAs for the dependent variables social connection and *kama muta* were used to examine the effect of live vs. recorded music. To address the third hypothesis that the salience of the coronavirus would facilitate a shared sense of connection, which in turn would lead to *kama muta*, we conducted a mediation analysis using the ‘mediation’ toolbox (Tingley et al., 2014).

For the exploratory results, we conducted backward selection approach multiple regressions and Kendall correlations to extensively examine the effect of all concert and personal variables on outcomes such as emotions, embodied reactions, and presence in addition to the main outcome variables of social connection and *kama muta*.

## Results

### Main Results

#### Empathic Concern, Social Connection, and *Kama Muta*

Based on previous research on *kama muta* as a social relational emotion, as well as previous findings relating empathic concern to feelings of being moved (Zickfeld et al., 2017), we hypothesized that *kama muta* and empathic concern would be related and that empathic concern would predict *kama muta*. Empathic concern, *kama muta*, and social connection were correlated using Pearson’s correlation and false discovery rate was controlled with the BH method (Benjamini and Hochberg, 1995). The sample was *n* = 300 for these analyses because 7 participants did not respond to the empathic concern scale. Social connection, *kama muta*, and empathic concern were all correlated [SC × KM: *r*(298) = 0.60, *p* < 0.001; EC × SC: *r*(298) = 0.16, *p* = 0.005; EC × KM: *r*(298) = 0.29, *p* < 0.001].

**FIGURE 1:**
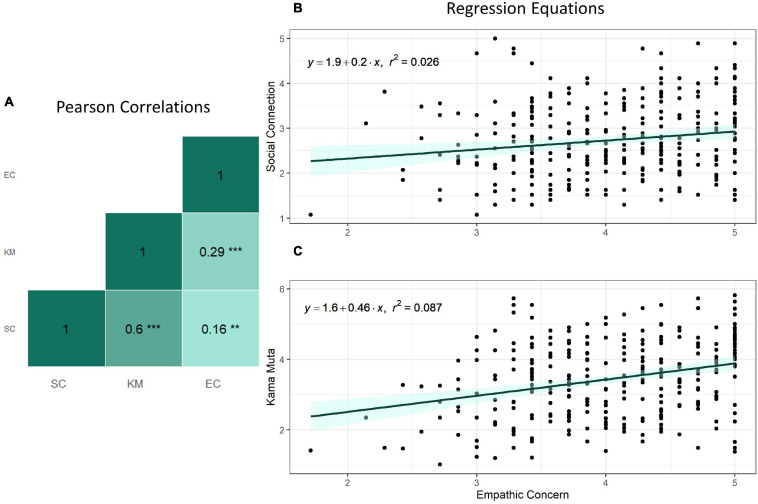
The relations between empathic concern, social connection, and *kama muta* with **(A)** correlations and regression equations of **(B)** social connection and **(C)** *kama muta*. **p < 0.01, ***p < 0.001.

Simple linear regressions were calculated to predict social connection and *kama muta* based on empathic concern. Empathic concern significantly predicted social connection and *kama muta* [social connection: β = 0.20, *R*^2^ = 0.026, *F*(1, 298) = 7.99, *p* = 0.005; *kama muta*: β = 0.46, *R*^2^ = 0.087, *F*(1, 298) = 28.27, *p* < 0.001] (see Figure 1).

#### Live vs. Pre-recorded

**FIGURE 2:**
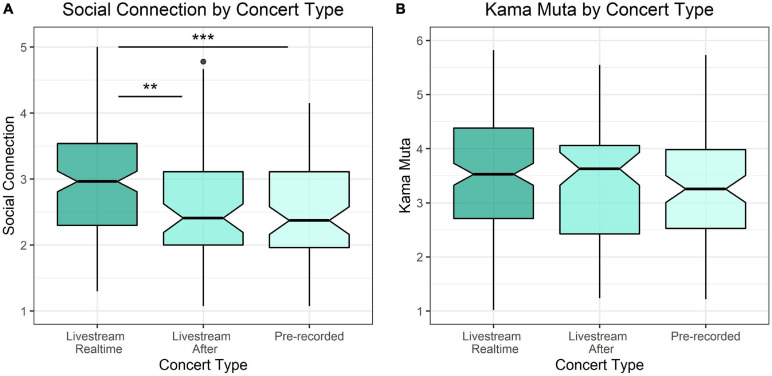
The effect of concert liveness on **(A)** social connection and **(B)** *kama muta*. **p < 0.01 and ***p < 0.001.

We hypothesized that live concerts would produce more social connection and *kama muta* than pre-recorded concerts or live-streamed concerts that were viewed after they were streamed. To test this hypothesis, a one-way ANOVA was fit to examine the effect of concert type on social connection and *kama muta* separately. Participants who were unsure of the concert type were excluded for this analysis (*n* = 8) therefore this analysis was conducted on a sample of *n* = 299. Concert type significantly affected social connection [*F*(2, 296) = 11.3, η*^2^* = 0.071, *p* < 0.001], but not *kama muta* [*F*(2, 326) = 1.61, η*^2^* = 0.010, *p* = 0.20] (see Figure 2). A Tukey *post-hoc* test revealed that livestreamed concerts resulted in more social connection (*M* = 2.97, *SD* = 0.84) than pre-recorded concerts (average = 2.50, *SD* = 0.74, *p* < 0.001) and live-streamed concerts watched after they were streamed (*M* = 2.54, *SD* = 0.82, *p* = 0.001). There was no difference in social connection between a pre-recorded concert and a live-streamed concert watched after it had been streamed (*p* = 0.94).

To ensure that this effect was not driven by whether participants were in the presence of others in the same physical space, we examined if there were any differences across concert types. There was no significant difference between the number of participants viewing with others across concert types [*χ*^2^(2) = 3.74, *p* = 0.15].

#### Salience of the Coronavirus

**FIGURE 3:**
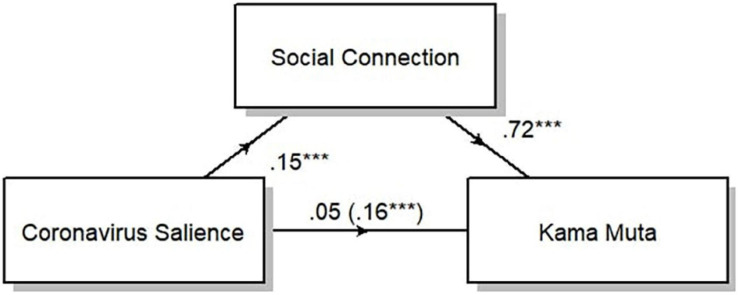
Social connection completely mediates the effect of coronavirus salience on *kama muta.* The diagram displays the average direct effects (0.05), the total effect (0.16), the effect of coronavirus salience on social connection (0.15), and the effect of social connection on *kama muta* (0.72). ***p < 0.001.

We hypothesized that when the coronavirus was salient during concerts, the audience would feel socially connected, which in turn would produce *kama muta*. To test this hypothesis, we conducted a mediation analysis that included all participants who responded to all items of social connection and *kama muta* including the item on the salience of the coronavirus (*n* = 307). The mediation analysis revealed that there was a significant effect of the independent variable (coronavirus salience) on both the dependent variable (*kama muta*) and the mediating variable (social connection). When the mediating variable was included in the linear regression of the independent and dependent variables, the effect of the independent variable disappeared. This means that the effect of coronavirus salience on *kama muta* was completely mediated by social connection (Figure 3). The regression coefficient between coronavirus salience and *kama muta* was significant (0.16, *p* < 0.001). The indirect effect was (0.15)^∗^(0.72) = 0.11 and the significance was tested using bootstrapping procedures (1000 samples) in the ‘mediation’ library for causal mediation analysis (Tingley et al., 2014). The 95% confidence interval of the indirect effect ranged from 0.06 to 0.16 and therefore was significant (*p* < 0.001). This result supports our hypothesis that participants would be more moved in concerts where there was more shared awareness of the pandemic because such concert experiences lead to feeling more connected.

We also tested the reverse mediation with *kama muta* as a mediator for the effect of coronavirus salience on social connection using the same methods described above. The effect of coronavirus salience on social connection was partially mediated by *kama muta* (see Supplementary Figure 1). The regression coefficient between coronavirus salience and social connection was significant (0.15, *p* < 0.001). The indirect effect was (0.16)^∗^(0.45) = 0.072. The 95% confidence interval of the indirect effect ranged from 0.04 to 0.11 and was significant (*p* < 0.001).

### Exploratory Results

We collected a large number of variables in the interest of understanding what other variables contribute to the social experience at concerts and in this section we conducted exploratory analyses to investigate how the other variables at concerts predicted the main outcomes. Multiple comparison corrections were performed and are described in the following sections.

#### Correlations

**FIGURE 4:**
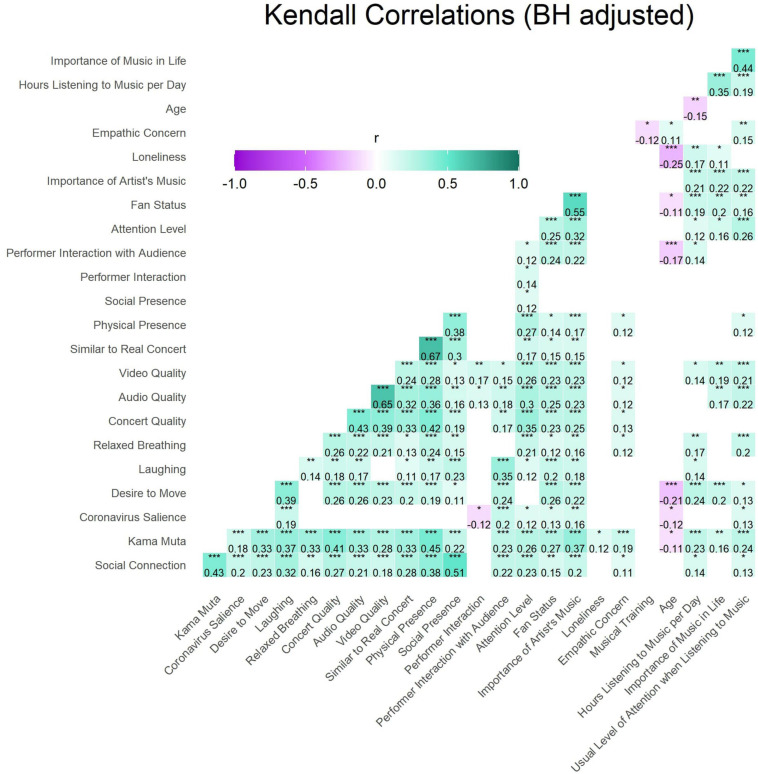
Kendall correlations were performed across variables with information on the individual participants’ characteristics (personal importance of music in the past 3 years, hours listening to music per day, age, empathic concern, average loneliness since the beginning of the pandemic, fan status, attention level during the concert, the number of other people present in the same space) concert characteristics (how much the performers interacted with the audience and each other, the social and physical presence experienced by the participant at the concert, participants’ appraisal of if it was similar to a real concert, perceived video and audio quality, overall concert quality, and salience of the coronavirus), and feelings or behaviors that the participant experienced at the concert (laughing out loud, desire to move, *kama muta*, and social connection). To control for false detection rate, the BH method was used. Only significant correlations are shown, thus the number of others present is not displayed because it had no significant correlations. Recall that the correlation between social connection and social presence is partially driven by the fact that these scales share two items. ^∗^*p* < 0.05, ^∗∗^*p* < 0.01, ^∗∗∗^*p* < 0.001.

Kendall correlations were performed to examine the influence of numeric variables of interest on a sample of participants who had responded to all numeric variables included in this analysis (*n* = 260). The *p*-values of the correlations were adjusted with the BH method for controlling the false discovery rate (Benjamini and Hochberg, 1995). Only significant correlations are displayed in the correlation matrix in Figure 4. All variables that were examined are included in Figure 4 except the quantity of other people watching with participants because this value was not correlated with any other variable.

The correlation figure reveals that most variables covaried moderately and positively with each other. In particular, our main variables of interest, *kama muta* and social connection, were related to most other variables. Performer interaction, musical training, and loneliness had the least relationships with other variables. Age had small negative relationships with desire to move and *kama muta*. The relations of personal and concert characteristics with emotional and bodily responses to the concert will be described further below.

#### Concert and Personal Characteristics

A backward selection approach to multiple regression was employed to determine which of the exploratory predictors best explained the outcome variables of social connection and *kama muta*. In addition to the main predictors of interest, concert liveness and coronavirus salience, we examined the additional influence of concert variables including the genre (e.g., pop, rock, and folk), concert setting (e.g., home, outdoors, and concert venue), web platform (e.g., Facebook, YouTube, etc.), perceived audio and video quality, shared country between the participant and the performer, and the performers’ level of interaction with other performers and with their audience. Additionally, we were interested in characteristics of the participant including the extent to which they were a fan of the artist, their average loneliness since the beginning of the pandemic, their attention level during the concert, whether they were watching with others in the same space, empathic concern, months of musical training, age, gender, region, hours listening to music per day, and the importance of music in their life in the past 3 years. To improve understanding of the effects of categorical predictors, results are presented in the form of ANCOVAs conducted in SPSS 26 with predictors selected from the backward regression. The model that best described the outcome of social connection explained 38% of the variance [*R^2^_*Adjusted*_* = 0.38, *F*(27, 229) = 6.70, *p* < 0.001] (see Table 1). The selection procedure resulted in genre, platform, concert liveness, whether others were watching in the same space and region as factors and covid salience, performer interaction with the audience, audio quality, attention level, fan status, empathy and how many hours they tend to listen to music per day as covariates. Three variables, namely whether others were watching in the same space, fan status, and number of hours of daily music listening, were not significant in the ANCOVA, so we excluded them from the final model. The final model explained 36% of the variance and the following predictors were retained in the final model in descending order of effect size (partial eta squared): genre, platform, attention level, concert liveness, region, audio quality, performer interaction with audience, empathy, and covid salience (see Table 1). We conducted *post-hoc* simple comparisons between levels of the factors with Bonferroni correction to understand which groups differed most from the others. We decided to report only results based on cells with more than 20 observations to increase the robustness of the findings. With these criteria, the pop genre (*n* = 80, *M*_e_^3^ = 2.42) differed significantly from the dance/electronic genre (*n* = 25, *M*_e_ = 2.95). Regarding platforms, Facebook (*n* = 76, *M*_e_ = 3.09) differed significantly from Other Website (*n* = 45, *M*_e_ = 2.50) and from YouTube (*n* = 134, *M*_e_ = 2.71). Regarding concert liveness, livestreamed concerts viewed in realtime (*n* = 154, *M*_e_ = 3.03) differed from livestreamed concerts viewed after they were streamed (*n* = 67, *M*_e_ = 2.67). Regarding regions, all significant differences involved Asia, but that group comprised only 10 participants.

**TABLE 1:** Significant predictors and effect size estimates for the ANCOVAs in order of descending effect size (partial eta-squared) explaining 1) social connection, 2) *kama muta*, 3) social presence, and 4) physical presence.

Predictor	β	*F*	*p*	η*^2^_*p*_*
**Social Connection**				
Genre	–	4.31	<0.001	0.100
Platform	–	3.59	0.001	0.095
Attention Level	0.217	19.90	<0.001	0.068
Concert Liveness	–	4.59	0.004	0.048
Region	–	4.14	0.007	0.043
Perceived Audio Quality	0.141	7.40	0.007	0.026
Performer Interaction with Audience	0.097	7.26	0.008	0.026
Empathic Concern	0.152	6.11	0.014	0.022
Coronavirus Salience	0.062	4.64	0.032	0.017
**Kama Muta**				
Empathic Concern	0.342	21.61	<0.001	0.069
Perceived Audio Quality	0.287	20.69	<0.001	0.066
Daily Hours Listening to Music	0.181	13.20	<0.001	0.043
Attention Level	0.189	11.02	0.001	0.036
Coronavirus Salience	0.104	10.39	0.001	0.034
Fan Status	0.129	7.84	0.005	0.026
Performer Interaction with Audience	0.093	5.18	0.024	0.017
**Social Presence**				
Platform	–	4.92	<0.001	0.122
Genre	–	3.48	0.002	0.069
Region	–	5.14	0.002	0.052
Audio Quality	0.207	9.09	0.003	0.031
Attention Level	0.191	8.959	0.003	0.031
**Physical Presence**				
Audio Quality	0.364	41.975	<0.001	0.129
Attention Level	0.281	28.649	<0.001	0.092
Platform	–	2.904	0.004	0.076
Loneliness	–0.127	10.079	0.002	0.034
Empathic Concern	0.161	5.692	0.018	0.020

*Parameter estimates of the coefficients (β) are provided for the numeric predictors (i.e. covariates), but not for factors to improve interpretation.*

The model that best described the outcome of *kama muta* explained 43% of the variance [*R^2^_*Adjusted*_* = 0.43, *F*(24, 232) = 9.09, *p* < 0.001] (see Table 1). Based on the backwards selection, we entered setting, platform, and gender as factors and the predictors covid salience, audio quality, performer interaction, performer interaction with the audience, attention level, fan status, empathic concern and how many hours the person typically listens to music per day. In successive analyses of covariance, first performer interaction, then gender, then platform and then setting failed to reach significance. These were therefore eliminated from the model. The final model explained 37% of the variance and included the continuous predictors empathic concern, audio quality, hours per day listening to music, attention level, covid salience, fan status and performer interacting with audience in descending order of effect size (partial eta squared), all with positive slopes.

#### Presence

Feelings of physical and social presence may be important contributors to the virtual concert experience. We aimed to examine how feelings of presence influence social connection and *kama muta* and additionally what concert characteristics contribute to feelings of presence. Therefore, we have chosen to explore the influence of presence on social connection and *kama muta* separately. The measure of social connection includes two items from the social presence scale therefore we removed these items from the measure of social connection for the purposes of examining the impact of presence on only the feelings and behaviors in the measure of social connection. First, we fit a simple linear regression to examine the effect of social presence on social connection and social presence explained 27% of the variance in social connection feelings and behaviors [β = 0.43, *R^2^_*Adjusted*_* = 0.27, *F*(1, 305) = 114.7, *p* < 0.001]. When physical presence was added as a predictor to the model with social presence, 33% of the variance in social connection was explained [social presence: β = 0.31, physical presence: β = 0.27, *R^2^_*Adjusted*_* = 0.33, *F*(2,304) = 75.8, *p* < 0.001].

**FIGURE 5:**
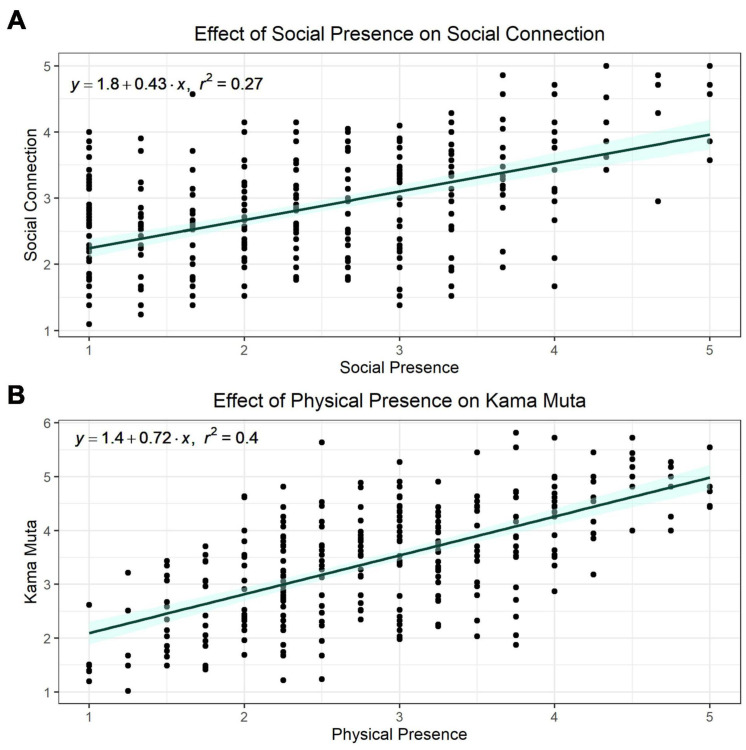
The effect of **(A)** social presence on social connection and **(B)** physical presence on *kama muta* and their regression equations.

We conducted a similar analysis with the outcome measure of *kama muta*. First, social presence was included as a predictor alone and social presence explained 11% of the variance in *kama muta* [β = 0.33, *R^2^_*Adjusted*_* = 0.11, *F*(1, 305) = 37.6, *p* < 0.001]. However, when physical presence was added as a predictor, the effect of social presence was no longer significant and together the predictors explained 39% of the variance in *kama muta* [physical presence: β = 0.71, social presence: β = 0.02, *R^2^_*Adjusted*_* = 0.39, *F*(2, 304) = 100.4, *p* < 0.001]. When examining just the effect of physical presence on *kama muta*, physical presence explained 40% of the variance in *kama muta* [β = 0.72, *R^2^_*Adjusted*_* = 0.40, *F*(1, 305) = 201.2, *p* < 0.001]. Together these results suggest that social presence had a large influence on feelings and behaviors of social connection and physical presence had a large influence on feelings and sensations of *kama muta* (see Figure 5).

We further explored the impact of the concert and personal characteristic predictors on social and physical presence to examine what elements of virtual concerts predict feelings of presence. We conducted a backward selection approach to examine which predictors lead to increased presence. The model that best described social presence explained 27% of the variance [*R^2^_*Adjusted*_* = 0.27, *F*(21, 241) = 5.69, *p* < 0.001] (see Table 1). The model selection resulted in a model with genre, platform, and region as factors and loneliness, audio quality, shared country with the performer, and attention level as predictors. Loneliness and shared country failed to reach the significance level. The final model explained 21% of the variance and contained platform, genre, region, audio quality and attention level in descending order of effect size. Simple comparisons between factor levels with at least 20 observations, Bonferroni adjusted, revealed that dance/electronic (*n* = 25, M_e_ = 3.50) produced more social presence than classical genres (*n* = 41, M_e_ = 2.64), pop (*n* = 82, M_e_ = 2.60) and metal (*n* = 24, M_e_ = 2.60). Regarding platforms, Facebook (*n* = 77, M_e_ = 3.20) led to more social presence than Other websites (*n* = 43, M_e_ = 2.42) and YouTube (*n* = 136, M_e_ = 2.45). Regarding regions, only Asia differed significantly from other regions, but it had only nine observations.

The model that best described physical presence explained 30% of the variance in physical presence [*R^2^_*Adjusted*_* = 0.30, *F*(11, 251) = 11.29, *p* < 0.001] (see Table 1). To best predict physical presence, we retained a model with platform as factor and audio quality, attention level, empathic concern, and loneliness as covariates. All remained significant in the ANCOVA, with audio quality being the best predictor, then attention level, platform, loneliness, and empathic concern (33% explained variance). The slopes of the covariates were positive, except for loneliness, which negatively predicted physical presence. Simple comparisons (Bonferroni corrected) revealed that Other websites (*n* = 42, M_e_ = 2.37) produced significantly less physical presence than YouTube (*n* = 133, M_e_ = 2.92) or Facebook (*n* = 76, M_e_ = 2.89).

#### Region and Salience of Coronavirus

The severity of the coronavirus varied across regions and it was particularly severe in parts of Chile and India during the recruitment phase of this experiment. The majority of participants came from Chile and 11 of 12 participants from Asia were based in India, therefore it was important to consider that differences in the salience of the coronavirus may be attributable to regional differences. Coronavirus salience was highest in Asia (*n* = 12, *M* = 3.83), then Chile (*n* = 211, *M* = 2.91), North America (*n* = 31, *M* = 3.03), and lowest in Europe (*n* = 95, *M* = 2.62). However, a one-way ANOVA indicated that there were no significant differences between regions on the measure of coronavirus salience [*F*(3, 345) = 2.56, *p* = 0.055].

#### Emotional and Embodied Reactions

**FIGURE 6:**
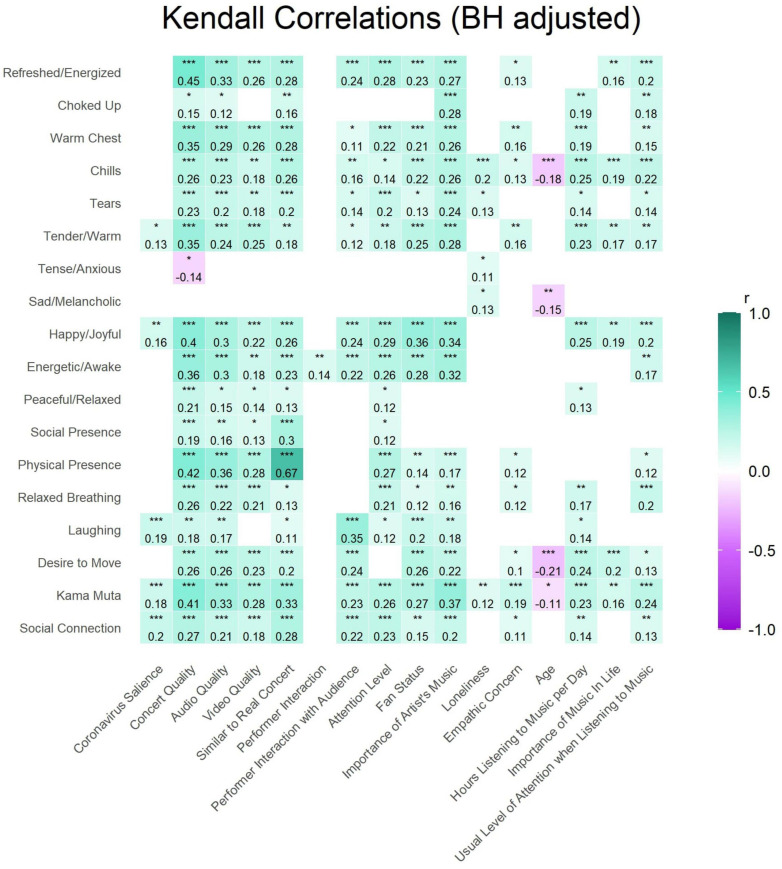
Kendall correlations with BH adjustment between concert and personal characteristics (*x*-axis) and outcomes of emotions and embodied reactions (*y*-axis). Note that some of the measures within the measure of *kama muta* are displayed here on the *y*-axis to show the correlation between specific embodied reactions and the predictor variables (i.e., refreshed/energized, choked up, warm chest, chills, and tears).

We asked participants to describe how the concert made them feel with a series of emotions. We also asked participants about their embodied sensations and actions such as the extent to which they experienced a desire to move, laughing out loud, and relaxed breathing (1 Not at all – 6 A lot). We conducted Kendall correlations between concert and personal characteristics and outcome variables of emotions and embodied reactions (see Figure 6). Concert and personal characteristics are displayed along the bottom of the correlation matrix while outcomes of feelings and embodied reactions are displayed along the left side.

Many concert and personal characteristics were correlated with embodied reactions and emotions. *Kama muta* was correlated with almost all concert and personal characteristics. Participants’ appraisals of the quality of the concert, audio, and video and how similar the virtual concert was to a real concert were positively correlated with most feelings and embodied reactions except for feeling tense/anxious and sad/melancholic with perceived concert quality being negatively correlated with feeling tense/anxious. The more performers interacted, the more energetic the participants felt, however, the extent to which performers interacted with the audience was correlated with many more variables including social connection, *kama muta*, embodied reactions of desire to move, laughing, tears, chills, warm chest, and feeling refreshed, happy, tender, and energetic. Fan-status and the importance of the artist’s music were correlated with both main outcomes and many embodied reactions and positive feelings. Loneliness which represents the average frequency that participants felt loneliness, lack of companionship, isolation, and anxiety since the beginning of the pandemic was correlated with feeling *kama muta*, feeling sad and anxious, and experiencing tears and chills. Age was negatively related to feeling *kama muta*, desire to move and chills. Items related to general music usage and importance were correlated with *kama muta*, desire to move, feeling happy and tender, and experiencing chills. Together the results suggest many concert and personal characteristics are related to emotions and embodied reactions.

The embodied reaction of desiring to move was not simply driven by the dance musical genre. A simple linear model revealed that there was an effect of genre on desire to move [*F*(6, 326) = 9.26, *p* < 0.001]. Specifically, all genres produced higher levels of desire to move compared to classical music (see Supplementary Table 1 and Figure 2A). Additionally, we examined which genres might lead to more relaxed breathing. A simple linear regression was not significant [*F*(6,326) = 2.03, *p* = 0.06] (see Supplementary Figure 2B).

#### Motivations for Attendance

**FIGURE 7:**
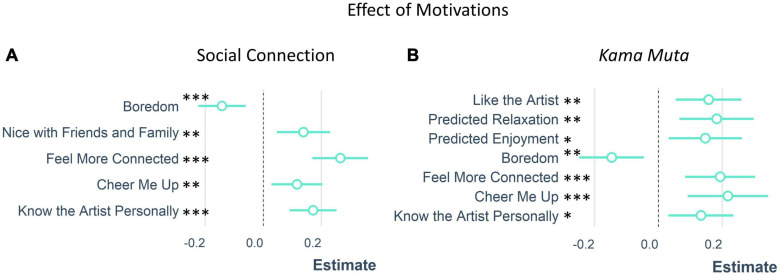
The effect of concert attendance motivations on **(A)** social connection and **(B)** kama muta.*p < 0.05, ^∗∗^*p* < 0.01, and ^∗∗∗^*p* < 0.001.

We asked people to report why they attended the virtual concerts and examined how their motivations for attendance affected social connection and *kama muta*. We conducted a multiple regression analysis to examine the effect of the motivation predictors on each outcome variable separately using a backward selection approach (see Figure 7 and Table 2). The model that best described the social connection explained 30% of the variance [*R^2^_*Adjusted*_* = 0.30, *F*(5, 301) = 26.9, *p* < 0.001] and contained the predictors that they thought it would be nice with friends and family, they wanted to feel more connected, cheer them up, they knew the artist personally, or they were bored. All predictors had positive slopes except for boredom which negatively predicted social connection. The model that best described *kama muta* explained 32% of the variance [*R^2^_*Adjusted*_* = 0.32, *F*(7, 299) = 21.5, *p* < 0.001] and contained the predictors that they liked the artist, predicted relaxation, predicted enjoyment, they wanted to feel more connected, they wanted to be cheered up, they knew the artist personally, or they were feeling bored. Again, all predictors had positive slopes except for boredom which negatively predicted *kama muta*.

**TABLE 2:** The effect of concert attendance motivations on **(A)** social connection and **(B)** *kama muta*.

Predictors	β	SE	p
**Social Connection**			
Boredom	–0.1	0.03	< 0.001***
Nice with Friends and Family	0.09	0.03	0.003**
Feel More Connected	0.17	0.03	< 0.001***
Cheer Me Up	0.09	0.03	0.01**
Know the Artist Personally	0.1	0.02	< 0.001***
**Kama muta**			
Like the Artist	0.14	0.05	0.003**
Predicted Relaxation	0.13	0.04	0.002**
Predicted Enjoyment	0.18	0.07	0.012*
Boredom	–0.11	0.04	0.005**
Feel More Connected	0.13	0.04	0.001***
Cheer Me Up	0.17	0.05	0.001***
Know the Artist Personally	0.08	0.03	0.01*

*p < 0.05, **p < 0.01, and ***p < 0.001.

#### Donations

We collected information on whether participants donated to the performers, to charities, or purchased tickets to the virtual concerts. Out of the *n* = 257 participants used in the models below, there were *n* = 25 participants who donated to the performers, *n* = 15 participants who donated to charity, and *n* = 22 participants who purchased a ticket.

We investigated what predictors were related to donating to the performers, donating to charities, and purchasing tickets with a backward selection approach using a logistic regression. In the full model, we included the same predictors as the models for the main outcome variables (see section “Social Media Recruitment”) and we additionally included whether artists asked for donations for themselves or charity, the measures of social connection, *kama muta*, physical and social presence, and the embodied reactions of desire to move, laughing, and relaxed breathing (see Table 3).

**TABLE 3:** Significant predictors and effect estimates for the backward selected model explaining donations to the performers, donations to charity, and ticket purchasing behavior.

Predictor	β	SE	P	OR	2.5% CI	97.5% CI
**Donations to Performers**						
Performer requested donations for themselves	2.32	0.67	0.001***	10.19	2.89	42.31
Concert Type: Pre-recorded vs. Livestreamed realtime	–2.69	0.89	0.003**	0.07	0.01	0.32
Platform: Other Website vs. Facebook	–3.53	1.37	0.01**	0.03	0	0.29
Shared Country	2.07	0.79	0.009**	7.91	1.86	43.73
Age	0.07	0.03	0.008**	1.07	1.02	1.13
**Donations to Charity**						
Performer Requested Donations to Charity	4.71	1.17	0***	110.95	15.89	1782.92
Audio Quality	–1.17	0.54	0.03*	0.31	0.09	0.82
Platform: Other Website vs. Facebook	–4.02	1.69	0.017*	0.02	0	0.29
Loneliness	–0.99	0.41	0.016*	0.37	0.14	0.76
**Purchased Tickets**						
Genre: Metal	3.11	1.4	0.027*	22.35	1.74	620.17
Genre: Pop	2.45	1.25	0.05*	11.59	1.35	267.05
Genre: Various	4.97	1.75	0.004**	144.74	5.97	8038.57
Platform: Other Website vs. Facebook	2.19	0.93	0.018*	8.96	1.6	65.25
Platform: Twitch vs. Facebook	3.42	1.59	0.031*	30.57	1.47	853.21
Platform: Zoom vs. Facebook	2.94	1.3	0.023*	18.91	1.63	306.57
Fan Status	0.96	0.48	0.042*	2.62	1.1	7.17
Usual Frequency of Concerts	0.83	0.35	0.017*	2.29	1.19	4.74
Predicted Enjoyment	1.18	0.59	0.046*	3.26	1.23	13.24

*p < 0.05, **p < 0.01, and ***p < 0.001.

Because conducting logistic regression requires that each category contains a response, we examined the cross tabulations of the data to ensure that each categorical variable contained at least one person who had donated in each category. For the model examining donations to performers, no one in the category of livestream viewed after had donated so these participants were re-coded to the pre-recorded concert category (*n* = 55). Additionally, we excluded participants from the gender categories of agender or preferred not to say (*n* = 4), from the genre category of various (*n* = 3), and from the platform category of TV (*n* = 1), Twitch (*n* = 5), and Vimeo (*n* = 1) because no one in these categories donated to performers. Region was excluded as a predictor from the analysis because none of the 25 participants from North America donated. For the model examining donations to charity, we excluded participants from the gender categories of agender or preferred not to say (*n* = 4), from the genre category of various (*n* = 3) and African-American genres (*n* = 13), and from the platform category of TV (*n* = 1), Instagram (*n* = 27), and Zoom (*n* = 9) because no one in these categories donated. Platform: Vimeo was excluded as well because the one participant who used Vimeo had donated to charity, therefore there were none who had not donated. Region was not included as a predictor again because no participants from Asia donated to charity (*n* = 10). Setting was also not included as a predictor because no participants who watched a concert with an outdoor setting donated to charity (*n* = 20).

Participants likely purchased tickets before the virtual concert, therefore for this model, we were interested in examining variables that would have been specified prior to the concert. Specifically, we examined the effects of motivations of attendance (see section “Motivations for Attendance” for the full list), concert type, genre, web platform, shared country, loneliness, fan-status, empathic concern, and age. Additionally, we were interested in exploring the effect of participants’ past concert attendance behavior with the variables of their usual frequency of attending concerts without the coronavirus restrictions, frequency of attending video concerts in the past month, and their frequency of attending streamed concerts within the past month (see Table 3).

### Efficacy of the Recruitment Methods

#### Industry and Artist Partnerships

The industry partnerships resulted in 5 recruitments and the artist partnerships resulted in 7 recruitments. One of our artist partners promoted through one of the industry partners; therefore, one participant was actually recruited through both an artist and industry partner so that the partnerships resulted in a total of 11 participants being recruited.

#### Social Media Recruitment

We also recruited participants with paid social media advertising through Facebook and Instagram (see Table 4).

**TABLE 4:** Social media recruitment costs and outcomes.

Country	Cost (£)	Reach	Clicks	n
United States	212	6940	25	11
Germany	143	6168	32	9
Norway	127	3362	26	74
Spain	85	8700	39	5
Chile	85	112,400	875	241
United Kingdom	170	7554	32	7
Canada	127	5930	32	22
India	85	189,100	572	12

*“Reach” is Facebook’s estimate of the number of people the ad was shown to. “Clicks” refers to the number of times people clicked on the link in the advertisement. “n” represents the number of participants recruited from each of the countries in which we advertised. Not all participants are shown in this table because some participants were recruited without social media advertising and some of these participants could have been recruited by other means as well.*

In Table 4 we show how much money was spent on recruitment per country, the “reach” which is Facebook’s estimate of the number of people the ad was shown to, the number of times people clicked on the link in the advertisement, and the number of participants we recruited from each of the countries in which we advertised. The total number of participants does not equal the number provided in this table.

## Discussion

### Summary

The aim of this research was to examine the aspects of virtual concerts that lead to feelings of social connectedness and *kama muta. Kama muta* is a Sanskrit term which translates to “moved by love” that is used to scientifically label the positive social-relational emotion often referred to as “being moved,” “feeling touched,” “heartwarming,” or “love” (Zickfeld et al., 2019). A central tenet of *kama muta* theory is that *kama muta* is evoked by a sudden increase in closeness or connectedness between people, but also between a person and, for example, a piece of music or the universe (Zickfeld et al., 2019).

During the coronavirus pandemic, governments introduced social distancing rules to prevent the spread of the virus and as a result, all live musical gatherings were canceled or moved online (Hacker et al., 2020). Participants who had recently watched virtual concerts were recruited to complete an online survey where they reported a variety of concert and personal characteristics in addition to social connectedness (collection of feelings and behaviors) and *kama muta* (measured with the short *kama muta* scale; Zickfeld et al., 2019). The main findings of the study were that (1) social connection and *kama muta* were related and predicted by empathic concern, (2) live concerts produced more social connection than pre-recorded concerts, and (3) the salience of the coronavirus predicted *kama muta* and this effect was completely mediated by social connection. This suggests that mentions or awareness of the pandemic increased *kama muta* by enhancing a sense of social connectedness. Exploratory analyses were conducted to understand what other concert and personal characteristics also predicted social connection and *kama muta* and the experience of social and physical presence (Makransky et al., 2017). Additionally, we investigated what predictors caused emotional and embodied reactions, what concert attendance motivations led to social connection and *kama muta*, and finally what predictors explained donation behavior. Together, these results suggest that organizers and performers can facilitate social connection and *kama muta* in their audiences by tuning various aspects of their virtual concerts.

### Main Results

As expected, social connection and *kama muta* were related and predicted by empathic concern in virtual concerts. Empathic concern is the tendency to experience the social emotions of sympathy and compassion for those experiencing negative events. Other recent research found that empathic concern predicted feeling more like the performer when listening to an audio recording of a concert, but not during a live concert (Leshem and Schober, 2020). Thus, greater levels of empathic concern may afford concert viewers a greater propensity for feeling connected in virtual environments. The empathic concern scale, devised nearly 40 years before the *kama muta* scale, includes the statement “I am often touched by things that I see happen” therefore it is logical that empathic concern is part of the larger construct of *kama muta* (Zickfeld et al., 2017). This claim is further supported by the present research which showed that empathic concern predicts *kama muta.* “Being moved” is one English term that describes *kama muta*, so it is perhaps no coincidence that *kama muta* and desire to move during the virtual concert were moderately correlated. One recent study showed that empathic concern was related to spontaneous movement during music listening, however, in the present study desire to move and empathic concern were only weakly correlated (Zelechowska et al., 2020). Empathic concern is also related to connectedness to nature (Di Fabio and Kenny, 2018) and reduced general prejudice (Levin et al., 2016) which suggests that this trait predisposes individuals to a variety of experiences of connectedness. In the real world, *kama muta* may be evoked by acts of rhythmic synchrony or exchanging substances such as food, however, in a virtual environment, exchanges can only occur virtually. Social connection was measured with virtual sharing, commenting, and liking behaviors along with the feelings of social presence and connectedness to the performer and other audience members. Social connection was positively correlated with *kama muta* which indicates that the intensification of communal sharing relationships can still occur in virtual environments through online social feelings and behaviors (Fiske et al., 2019).

The improved social connection during livestreamed concerts likely reflects how shared experiences produce feelings of togetherness (Peck and Shu, 2018; Miller, 2020). Previous research on how technologically mediated communication can foster social connection during the coronavirus pandemic suggests that shared online virtual experiences foster a sense of togetherness (Miller, 2020). While video conferencing was the most popular form of interaction during the pandemic, shared online virtual experiences such as virtual exercise classes or concerts were the second most popular form (Miller, 2020). Some social media platforms have begun to introduce features that make livestream replays seem more like they are occurring in realtime such as the “live chat replay” offered in YouTube and Facebook that displays audience members’ comments as they would have appeared during the live concert. Despite the presence of these features, audience members’ social experiences of livestream replays were not different from pre-recorded concerts. Contrary to our hypothesis, there was no effect of concert type on *kama muta*. It is possible that the closeness facilitated in live concerts is simply maintained rather than intensified by any indication of liveness, and thus this closeness would not have occurred with the suddenness required to elicit *kama muta*.

In related research, the frequency that participants felt lonely since the beginning of the pandemic was correlated with the frequency of viewing livestreams in realtime, but not those viewed after they were aired (Onderdijk et al., 2021), therefore participants may be using live virtual concerts to satisfy their missing social needs. Livestreamed concerts may be used strategically in times of social distancing to facilitate social connectedness and mitigate feelings of loneliness and its associated negative health consequences.

Greater awareness of the social-distancing restrictions of the coronavirus during the concert, which may have been facilitated when performers or audience members discussed the topic, resulted in greater social connectedness and *kama muta*. Social connection completely mediated the effect of the salience of the coronavirus on *kama muta*. We also tested the reverse mediation and found that *kama muta* partially mediated the effect of coronavirus salience on social connection, thus the complete mediation explains the directionality of the effect better. Together, these findings support the theoretical underpinnings of *kama muta* by reinforcing the importance of the temporal suddenness of closeness for eliciting this emotional experience (Zickfeld et al., 2019). However, the underlying mechanism cannot be fully elucidated in the present research. Participants in other research reported that a sense of comfort came from discussions that highlighted they were not alone in their experiences of loneliness and struggle during the pandemic (Miller, 2020). Therefore, this solidarity through shared hardship is one potential mechanism that could have fostered an intensified connectedness and subsequently *kama muta*. Coronavirus salience was positively correlated with the emotions of being happy/joyful and tender/warm and the embodied reaction of laughing out loud. Therefore, it is additionally possible that the artists and audiences discussed COVID-19 and social distancing in positive, humorous ways. Future research could manipulate the extent to which shared narratives are made salient in a concert using different strategies such as through joke telling or by fostering a sense of solidarity to better understand the mechanisms that cause a sudden onset of closeness and the socioemotional reaction of *kama muta*.

### Exploratory Results

We used correlations and backward selection multiple regression to explore the important variables for facilitating social connection and *kama muta.* Social connection was correlated with many variables including embodied reactions of desire to move, laughing, and relaxed breathing; concert characteristics of perceived concert quality, audio quality, video quality, similarity to a real concert, and amount of performer interaction with the audience; and participant characteristics of attention level, fan status, the importance of the performer’s music, and empathic concern. Interestingly social connection and *kama muta* were not correlated with performer interaction, suggesting that this is not an important component in experiencing social connectedness, and performer interaction was negatively correlated with coronavirus salience likely because concerts featuring much interaction between performers may have been recorded before the pandemic. However, supporting theories of the importance of social connectedness in eliciting *kama muta, kama muta* was correlated with all of the same variables correlated with social connection and additionally loneliness, and it was negatively correlated with age. Loneliness and desire to move were also negatively correlated with age which possibly reflects that older participants viewed concerts with genres that did not elicit the desire to move and that older participants may have had partners or families that prevented them from feeling lonely during the pandemic; thus they did not have the same predisposition to feeling a sudden intensification of connectedness that would produce *kama muta*. Loneliness may be associated with *kama muta* because the sudden contrast in low connectedness before and greater connectedness during the concert may make individuals more likely to experience intensifications of the communal sharing relationships (Fiske et al., 2019).

When examining the regression models, the important predictors for both social connection and *kama muta* were perceived audio quality, coronavirus salience, performer interaction with the audience, attention level, and empathic concern. Audio quality was also an important predictor of both social and physical presence therefore concert organizers and viewers alike should aim to achieve the best possible audio quality to optimize virtual concert experiences. This finding is also contrary to previous research showing that audio quality was not an important predictor in facilitating music video enjoyment, however, it is possible that the constraints of the pandemic resulted in even lower qualities than those examined in previous research or that enjoyment may be distinct from connectedness and *kama muta* (Schoeffler and Herre, 2014). Similarly, attention levels were important predictors of social and physical presence, perhaps because attention is a prerequisite for feelings of presence and social experiences, so concert organizers could aim to capture audiences’ attention and viewers should pay attention to receive the full benefit of virtual concerts. Performers may be able to capture audiences’ attentions by interacting with audiences even in the virtual concert environment which will also enhance audience members experiences of social connection and *kama muta.* Importantly, even using the backward selection procedure, coronavirus salience emerged as an important predictor of both social connection and *kama muta* which means that performers and audiences could foster these emotions by discussing topics of shared struggles.

There were several variables that predicted social connection, but not *kama muta*, including platform, genre, region, and concert liveness. Facebook produced more social connection and social presence than YouTube or other websites that were not major social media sites. Facebook alerts users when a friend is interested in an event or when another friend is watching the same live event which might foster a sense of togetherness more than seeing other users who are strangers engaging in concerts through the simple live chat function of YouTube. Interestingly, both Facebook and YouTube produced more physical presence than other websites. Concert organizers could leverage these features of Facebook to facilitate more social connection and presence in their livestreams.

The genre of dance/electronic music also produced more social connection than pop. Similarly, dance produced more social presence than classical, pop, and metal genres. Dance music typically elicits movement from listeners (Dyck et al., 2013) and despite listening alone, it is possible that moving with the performers may have resulted in enhanced bonding as research shows that synchronized movement fosters social bonding, even through video (Launay et al., 2014). Motor entrainment with the music or with performers’ movements, or simple action observation of performers’ movements, could have activated kinesthetic empathic processes and made participants feel more socially connected (Reason and Reynolds, 2010).

A very interesting result from the exploratory analyses was that (with the social presence items removed from the social connection measure) social presence predominantly predicted social connection (*R^2^_*Adjusted*_* = 0.27) and physical presence predominantly predicted *kama muta* (*R^2^_*Adjusted*_* = 0.40). The multimodal social presence subscale measures whether participants feel the presence of others and whether they feel as if others are aware of their own presence (Makransky et al., 2017). The physical presence subscale measures how real the environment feels, their immersion in the environment or lack of attention to the real world, and how much they feel a sense of control in the virtual environment (Makransky et al., 2017). Feeling social presence is likely an important pre-cursor to feeling social connection. Interestingly, in other related work where feelings of presence were directly manipulated, social presence predicted more social connection to the audience and physical presence predicted more social connection to both the performers and audience (Onderdijk, Swarbrick et al., 2019). Therefore, feeling social connection with the performer specifically and physically present in the virtual environment may be important for eliciting *kama muta* possibly because the important communal sharing relationship at virtual concerts is between the performer, their music, and the individual audience members rather than between the audience members themselves. This supports the theory on fandom as a continuous search for *kama muta* experiences elicited by performers and their music (Fiske et al., 2019). Fan-status was the only unique predictor of *kama muta* as it did not predict social connection or presence. Listeners aim to repeat *kama muta* experiences evoked from performers’ music by continuing to engage in their media through music listening, sharing and interacting on social media, and by attending concerts (Bannister, 2019). Fandom is essentially devotion to celebrity personae through parasocial interactions and the experiences of *kama muta* may increase this devotion (Brown, 2015).

We examined how concert attendance motivations contributed to the experience of social connection and *kama muta.* Participants who were attending simply from boredom experienced less social connection and *kama muta.* Participants who wanted to feel more connected, who wanted to be cheered up, and who knew the artist personally were more likely to experience social connection and *kama muta.* Doing something nice with friends and family was a unique predictor for the model predicting social connection while unique to *kama muta* were the motivations of predicted relaxation and enjoyment. These results should be interpreted with caution because these motivations were reported retrospectively so it is possible that the concert experience changed their memories of why they initially wanted to attend the concerts. However, together these results suggest that participants’ intentions and predictions can modify the social experience of virtual concerts.

Concert attendance is often cited as a favorite musical experience (Krause et al., 2020). Live music attendance is beneficial for health and wellbeing (Fancourt and Steptoe, 2018) and offers concert-goers the ability to connect with other fans (Dearn and Price, 2016) and to interact, albeit parasocially, with performers (Kurtin et al., 2019). Ticket sales for concerts were a vital source of income for musicians before the pandemic due to increased accessibility to pirated music and steadily declining revenues from streaming (Naveed et al., 2017). While cost may not influence decisions to attend real live concerts (Brown and Knox, 2017), virtual concerts may not be granted the same generosity. Therefore, it is important to examine the variables that lead to donations and ticket purchasing for virtual concerts.

To understand ticket purchasing behavior we conducted a backward selection approach to identify predictors that would have led to purchasing tickets before the concert. Significant predictors of ticket purchasing behavior were the genres of metal, pop, and various, the platforms of other website, Twitch, and Zoom compared to Facebook, attendance motivations of liking the artist and predicting enjoyment, fan-status, and usual frequency of concerts (before the pandemic). To understand donations to the performers and charities we conducted a similar approach, but by including those variables that were measured based on the experience of the concert including their experiences of social connection and *kama muta*. Significant predictors for donations to the performers were if the performer asked for donations for themselves, being a livestreamed concert in real time as opposed to a pre-recorded concert, using the platform of Facebook as compared to Instagram or another website, being in the same country as the performer, being less lonely, and being older. Significant predictors for donations to charity were if the performer asked for donations to charities, livestreamed concert (as compared to pre-recorded), less audio quality, more laughing, less loneliness, and being on the platform of Facebook as opposed to another website or YouTube.

Together these results show that to encourage ticket purchasing, performers could host concerts on Twitch, Zoom, and other websites and focus on selling tickets to their existing fanbase. Participants who attended more concerts before the pandemic likely understood the value of these concerts and therefore did not mind spending money on tickets for virtual concerts, because it was a pre-existing expense. To facilitate more donations, it is important that performers actually request them for themselves or for charities. Performers have a privileged space in peoples’ lives where audiences are receptive when they advocate for causes or for themselves. The liveness of the concert was important in facilitating donations as well, either because people feel more accountable or generous when at a live event or because more pre-recorded concerts occurred before the pandemic. People who are older likely have more money to donate to performers which explains the positive association with age. Interestingly, being lonelier led to less donations to the performer and charities possibly because these individuals were struggling financially themselves or because their lack of social support made it challenging for them to give financial support. Sharing a country with the performer may have made it easier to donate because of shared banking systems and currency. Facebook introduced the software infrastructure allowing audience members to easily donate to charities and to performers directly within their platform. More laughing out loud and lower audio quality were also predictors of donations to charities; however, these predictors are more challenging to interpret. Laughter was correlated with fan-status (0.22), coronavirus salience (0.24) and performer interaction with the audience (0.39) therefore it is possible that when a loved performer interacted with the audience, perhaps by encouraging donations with some joking about the coronavirus, this could have made participants laugh out loud and inspire donations to charity. The finding that higher audio quality predicted less donations to charity is peculiar, especially given that audio quality was found to be a predictor of social connection and *kama muta*. It is possible that the higher quality made participants experience more physical presence and thus helped them forget about the coronavirus pandemic and the importance of donating. Alternatively, it could be that if people are going to donate, they choose between donating to the performer or charities and if the audio quality is poor they are more likely to donate to a charity than the performer. Future research should aim to test these exploratory findings directly.

### Limitations and Future Directions

While we made great efforts to sample from different regions and concert types, our final sample is certainly not representative of any region or of the totality of virtual concerts. This is a limitation when it comes to interpreting regional differences. Furthermore, while the mediation effect for our third hypothesis is consistent with our expectations, we cannot establish causality in a cross-sectional study.

We reported a large number of tests which greatly increases the risk of a type I error, and thus especially the exploratory results should be interpreted with caution. Additionally, to effectively explore the data we conducted regressions with stepwise approaches combined with theoretical knowledge of the predictors. However, we recognize that future research should aim to experimentally manipulate and test the effects of variables that predict social connection and *kama muta* from this study.

Methodologically, re-coding of genre and setting relied heavily on experimenter’s judgments. It is possible that some of the coding of genres and settings may have resulted in incorrect classification. We recognize that there are artists who may cross several genre categories; therefore, the analysis with genre should be interpreted carefully. We additionally wish to recognize that these genre classifications do not provide a nuanced awareness of the impact of genres, and we encourage future researchers to take an experimental approach by directly manipulating musical genre. Further reflection on the types of responses we expected could have allowed us to provide closed response options to participants which could have improved survey completion rate and improved validity (for example, providing a list of genres and settings that participants could choose from). However, we now have a richer dataset than we would have had if we had provided forced choice instead of free text response options. Other researchers are now able to use this data to ask their own questions. For example, a researcher could explore whether religious music, or settings facilitated greater social connection and *kama muta.* The detail provided by the free text options will allow researchers to explore this question and more.

Coronavirus salience was measured with a single item that aimed to measure how much participants were aware of the coronavirus during the concert. Examples of whether the performer or audience members mentioned the coronavirus were provided. However, there are other ways in which the participants may have been aware of the coronavirus such as their personal situation (e.g., quarantine status, at-risk group), other media and news, or if they were worrying about loved ones that our survey did not measure. Future work should aim to measure awareness of troublesome situations or should aim to experimentally manipulate participants’ awareness.

We included all participants who reported recently watching a concert for more than 15 minutes. However, information on how recently they had viewed the concert was not collected. We also did not provide a rule for how recently participants needed to have watched the concert to be included in the experiment. Therefore, participants’ responses were possibly dependent on the accuracy of their memories. Future research could explicitly collect this information or directly manipulate concert experiences to avoid this confound.

Participants’ responses to the question on the size of the audience indicated that this question was unclear because some responded with information from the audience that was present during the filming of the concert video if their concert video had an audience, while we were trying to measure the size of the virtual audience who was simultaneously streaming with participants. Future research should consider asking specifically about the number of other people streaming the concert if that information is available or the number of people in the video’s audience depending on what measure they wish to collect.

Globalization has provided us with many tools to connect with people across the world. The same phenomenon that makes it more likely for pandemics to occur also allows researchers to collaborate internationally and recruit larger sample sizes. However, as researchers, we should also carefully consider the impact that our recruitment methods have not only on the data we collect but also the people we collect from. The advertising we used inadvertently caused a bias in the dataset because the advertiser had varied fees across different countries. We encourage future researchers to think critically with an intersectional and equitable lens to ensure they are engaging in ethical recruitment. We hope that by being transparent with our recruitment methods and their efficacy, we are contributing to the mandate of open science.

## Conclusion

We aimed to examine the variables that produce social connection and *kama muta* in virtual concerts during the coronavirus pandemic. We found that (1) social connection and *kama muta* were related and predicted by empathic concern, (2) live as compared to pre-recorded virtual concerts facilitated more social connection, and (3) greater salience of the coronavirus pandemic produced more *kama muta* and this effect was completely mediated by social connection. This research contributes to existing research on the social-relational emotion often called “being moved” by showing the importance of perceived physical presence in evoking *kama muta* and perceived social presence in evoking social connection in virtual environments. These results provide actionable guidance for performers and virtual concert organizers on how they can facilitate social connection and *kama muta* among their audiences and encourage ticket purchasing and donations.

## Data Availability Statement

The datasets presented in this study can be found in an Open Science Foundation online repository accessible here: https://osf.io/skg7h/.

## Ethics Statement

The studies involving human participants were reviewed and approved by the Research Ethics Committee, Department of Psychology, University of Oslo. The participants provided their written informed consent to participate in this study.

## Author Contributions

DS and JV designed the experiment and DS, JV, and BS developed the questionnaire. DS collected and analyzed the data and conducted statistical analyses. BS conducted the analyses in SPSS. DS wrote the majority of the manuscript and JV and NG contributed to the introduction. DS, JV, NG, and BS edited and approved the final manuscript. All authors contributed to the research and approved the submitted version.

## Conflict of Interest

The authors declare that the research was conducted in the absence of any commercial or financial relationships that could be construed as a potential conflict of interest.

## Footnotes

1 https://uio.no/ritmo/quarantineconcerts

2 https://osf.io/skg7h/

3 M_e_ represents estimated marginal means.

## Supplementary Material

Supplementary 1 Questionnaire in **(A)** English, **(B)** Norwegian, German, and Spanish.

Supplementary 2 Pre-processing details.
