# Supplementary material for: Corona Concerts: The Effect of Virtual Concert Characteristics on Social Connection and Kama Muta
Source: Front Psychol. 2021 Jun 22;12:648448. doi: 10.3389/fpsyg.2021.648448 (PMC8260031; doi:10.3389/fpsyg.2021.648448)
Supplement: Supplementary 1 — Questionnaire in (A) English, (B) Norwegian, German, and Spanish. [file Data_Sheet_1.DOCX]

Supplementary Material

# S1: Questionnaire & Translations

## A) English

Study title: Online concert experiences under conditions of physical distancing

(Note: you can select German, Norwegian, or Spanish versions of the survey from the menu in the upper right-hand corner) (Nota: usted puede seleccionar una versión en español de la encuesta desde el menú en la esquina superior derecha)

**Informed Consent**

**Background and purpose** This is an invitation to participate in a research study about online concert experiences under conditions of physical distancing. We are interested in your experiences during the last online concert you experienced. This research is conducted by a team at the University of Oslo. You may contact Dana Swarbrick, dana.swarbrick@imv.uio.no, with any questions and comments.

**What does the study entail?** During the study, you will be asked to complete several questions with regard to the last online concert you experienced. We will ask you about your enjoyment of the music and your impressions of the situation. The study takes about 10 to 15 minutes to complete. In the end, you will be able to take part in a lottery in which you can win 1 out of 20 vouchers (20 USD value). The information provided with regard to the lottery cannot be linked to your present responses.

**Potential advantages and disadvantages** The study includes no known potential disadvantages.

**What will happen to the information about you?** The samples and data that are registered about you will only be used in accordance with the purpose of the study as described above. All the data and samples will be processed without name, ID number or other directly recognisable type of information. It will not be possible to identify you in the results of the study when these are published.

**Voluntary participation** Participation in the study is voluntary. In order to participate you need to be 18 or older. You can withdraw your consent to participate in the study at any time and without stating any particular reason. This will not have any consequences for your further treatment. If you wish to participate, confirm your consent below before proceeding.

**Privacy** Information that is retained about you are only the answers that you give in the questionnaire. No identifiable information, such as IP address, is saved.

**Releasing material and data to other parties** Your answers are merged with the answers of the other participants in a large database; your answers cannot be traced back to you. This database will be posted in an open repository upon completion of the project, which is recommended best practice in any psychological research.

**Funding and the role of University of Oslo** The study is funded by research grants from the Department of Psychology and the Faculty of Humanities at the University of Oslo, Norway.

**Information about the outcome of the study** You are entitled to receive information about the outcome/result of the study. Please contact the research team to do so.

**I have read and agree with the terms above**

- Yes (1)

Information

Welcome to the “**Online concert experiences under conditions of physical distancing”** study. We aim to understand what contributes to the experience of an online concert. The following survey should take approximately 10-15 minutes to complete

Q1 Have you recently watched an online concert for more than 15 minutes?

- Yes (1)
- No (2)

Display This Question:

If Have you recently watched an online concert for more than 15 minutes? = No

Q2
In order for you to answer these questions it is important that you have adequate experience with online concerts. Please spend at least 15 minutes watching an online concert of your choice before answering this survey.

Skip To: End of Survey If In order for you to answer these questions it is important that you have adequate experience with... Is Displayed

age How old are you?

________________________________________________________________

Display This Question:

If If How old are you?<o:p></o:p> Text Response Is Less Than 18

Not_old We are sorry. If you are under 18 years old, ethical laws state that you do not have the ability to provide informed consent.

Skip To: End of Survey If We are sorry. If you are under 18 years old, ethical laws state that you do not have the ability... Is Displayed

gender What is your gender?

- Woman (1)
- Man (2)
- Prefer not to say (3)
- Prefer to self-describe (4) ________________________________________________

country In which country do you reside?

________________________________________________________________

End of Block: Block 1

Start of Block: Block 2

Q6 How often do you attend live musical concerts usually (without social distancing measures)?

- Never (1)
- Less than once a month (2)
- Once or twice a month (3)
- About every week or every other week (4)
- More than once a week (5)

Q7 How often have you watched video-recorded concerts in the past month?

- Never (1)
- Less than once a month (2)
- Once or twice a month (3)
- About every week or every other week (4)
- More than once a week (5)

Q8 How often have you watched live-streamed concerts in the past month?

- Never (1)
- Less than once a month (2)
- Once or twice a month (3)
- About every week or every other week (4)
- More than once a week (5)

| Page Break |  |
| --- | --- |

Copy_url Please copy and paste the link/URL of where you accessed the last concert you listened to or watched online for at least 15 minutes:

________________________________________________________________

No_url

- I don't have a link/URL (1)

Display This Question:

If = I don't have a link/URL

Q9 Please report the platform you viewed the concert on (e.g. Facebook, YouTube)

________________________________________________________________

Q10 How much of the concert did you watch (minutes)?

________________________________________________________________

Q11 What kind of concert did you watch?

- I watched a live-streamed concert in real-time (as it happened) (1)
- I watched a live-streamed concert, not in real-time (after the concert had ended) (2)
- I watched a pre-recorded concert (3)
- Not sure (4)

Q13 What is the name of the artist(s)? (e.g. Cher, The Who, Berlin Philharmonics)

________________________________________________________________

Q14 What genre was the concert?

________________________________________________________________

Q15 What was the location/setting of the concert? (e.g. kitchen, concert venue)

________________________________________________________________

Q16 In which country did the concert take place?

________________________________________________________________

Q17 Are you a fan of the artist?

- 1 (Not at all) (1)
- 2 (2)
- 3 (3)
- 4 (4)
- 5 (Totally) (5)

Q18 How important is the music performed by this artist for you? (e.g. one of their songs was important during a significant time of your life)

- 1 (Not at all) (1)
- 2 (2)
- 3 (3)
- 4 (4)
- 5 (Extremely) (5)

Q19 Please select the equipment you used to listen and view the concert in the two questions below:

Q20 Please check off the screen you saw the concert on:

- Phone screen (1)
- Tablet (2)
- Laptop (3)
- Desktop screen (4)
- Big TV screen (5)
- Large projection (6)
- Other: Please describe (7) ________________________________________________

Q21 Please check off the audio equipment you used:

- Simple headphones/earplugs (1)
- High-quality headphones/earplugs (2)
- Built-in speakers (3)
- External speakers (4)
- High-quality speakers (5)
- Other: Please describe (6) ________________________________________________

Q22 What was your level of attention when you were watching the concert?

- 1 (Background only) (1)
- 2 (2)
- 3 (3)
- 4 (4)
- 5 (Total concentration) (5)

| Page Break |  |
| --- | --- |

Q23 How often did the performers interact with each other?

- There was only one performer (1)
- 1 (Not often) (2)
- 2 (3)
- 3 (4)
- 4 (5)
- 5 (Very often) (6)

Q24 How often did the performers interact with the audience?

- Never (1)
- Once or twice (2)
- Occasionally (3)
- Frequently (4)
- Very often (5)

Q25 How much did you notice the presence of other audience members?

- 1 (Not at all) (1)
- 2 (2)
- 3 (3)
- 4 (4)
- 5 (Very much) (5)

Q26 Did you know any other people who were in the audience?

- Yes (please indicate approximately how many people you knew in the audience) (1) ________________________________________________
- No (2)

Q27 How salient were the coronavirus-induced social distancing circumstances (e.g. did the performer or audience members discuss the topic or encourage donations)?

- 1 (Not at all salient) (1)
- 2 (2)
- 3 (3)
- 4 (4)
- 5 (Very salient) (5)

Q28 Please describe in your own words how watching the concert made you feel:

________________________________________________________________

Q29 How connected did you feel to the performer(s)?

- 1 (Not at all) (1)
- 2 (2)
- 3 (3)
- 4 (4)
- 5 (Extremely) (5)

Q30 How connected did you feel to the other audience members?

- 1 (Not at all) (1)
- 2 (2)
- 3 (3)
- 4 (4)
- 5 (Extremely) (5)

Q31 Were other people watching the concert with you in the same physical space?

- Yes (please indicate how many) (1) ________________________________________________
- No (2)

Q32 To what extent did you feel that you shared emotions with other audience members?

- 1 (Not at all) (1)
- 2 (2)
- 3 (3)
- 4 (4)
- 5 (Extremely) (5)

| Page Break |  |
| --- | --- |

Feel Please describe how the concert made you feel:

Q33 Moved/touched

- 1 (Not at all) (1)
- 2 (2)
- 3 (3)
- 4 (4)
- 5 (Extremely) (5)

Q34 Peaceful/relaxed

- 1 (Not at all) (1)
- 2 (2)
- 3 (3)
- 4 (4)
- 5 (Extremely) (5)

Q35 Energetic/awake

- 1 (Not at all) (1)
- 2 (2)
- 3 (3)
- 4 (4)
- 5 (Extremely) (5)

Q36 Happy/joyful

- 1 (Not at all) (1)
- 2 (2)
- 3 (3)
- 4 (4)
- 5 (Extremely) (5)

Q37 Sad/melancholic

- 1 (Not at all) (1)
- 2 (2)
- 3 (3)
- 4 (4)
- 5 (Extremely) (5)

Q38 Tense/anxious

- 1 (Not at all) (1)
- 2 (2)
- 3 (3)
- 4 (4)
- 5 (Extremely) (5)

Q39 Tender/warm

- 1 (Not at all) (1)
- 2 (2)
- 3 (3)
- 4 (4)
- 5 (Extremely) (5)

KAMMUS-S Please indicate whether you experienced any of the following sensations, feelings, or actions during the concert, and if so, to what extent:

Q40 Tears

- 1 (Not at all) (1)
- 2 (2)
- 3 (3)
- 4 (4)
- 5 (5)
- 6 (A lot) (6)

Q41 Chills or shivers

- 1 (Not at all) (1)
- 2 (2)
- 3 (3)
- 4 (4)
- 5 (5)
- 6 (A lot) (6)

Q42 A warm feeling in the center of the chest

- 1 (Not at all) (1)
- 2 (2)
- 3 (3)
- 4 (4)
- 5 (5)
- 6 (A lot) (6)

Q43 Choked up

- 1 (Not at all) (1)
- 2 (2)
- 3 (3)
- 4 (4)
- 5 (5)
- 6 (A lot) (6)

Q44 Refreshed, energized, or exhilarated

- 1 (Not at all) (1)
- 2 (2)
- 3 (3)
- 4 (4)
- 5 (5)
- 6 (A lot) (6)

Q45 Desire to move your body such as toe-tapping, head-bobbing, or dancing

- 1 (Not at all) (1)
- 2 (2)
- 3 (3)
- 4 (4)
- 5 (5)
- 6 (A lot) (6)

Q46 Laughing out loud

- 1 (Not at all) (1)
- 2 (2)
- 3 (3)
- 4 (4)
- 5 (5)
- 6 (A lot) (6)

Q47 Relaxed breathing

- 1 (Not at all) (1)
- 2 (2)
- 3 (3)
- 4 (4)
- 5 (5)
- 6 (A lot) (6)

Q48 Other (please describe)

________________________________________________________________

Stat Please rate to what extent each of the following statements are true:

Q49 I felt an extraordinary feeling of welcoming or being welcomed.

- 1 (Not at all) (1)
- 2 (2)
- 3 (3)
- 4 (4)
- 5 (5)
- 6 (A lot) (6)

Q50 I observed an exceptional sense of closeness appear.

- 1 (Not at all) (1)
- 2 (2)
- 3 (3)
- 4 (4)
- 5 (5)
- 6 (A lot) (6)

Q51 I had positive feelings.

- 1 (Not at all) (1)
- 2 (2)
- 3 (3)
- 4 (4)
- 5 (5)
- 6 (A lot) (6)

Feel Please indicate whether you had each of the following feelings just afterwards, and if so, to what extent:

Q52 I felt like telling someone how much I care about them.

- 1 (Not at all) (1)
- 2 (2)
- 3 (3)
- 4 (4)
- 5 (5)
- 6 (A lot) (6)

Q53 I wanted to hug someone.

- 1 (Not at all) (1)
- 2 (2)
- 3 (3)
- 4 (4)
- 5 (5)
- 6 (A lot) (6)

| Page Break |  |
| --- | --- |

Loneliness Since the start of the social distancing measures, how often have you felt the following:

Q54 Loneliness

- 1 (Hardly ever) (1)
- 2 (2)
- 3 (Some of the time) (3)
- 4 (4)
- 5 (Often) (5)

Q55 Lack of companionship

- 1 (Hardly ever) (1)
- 2 (2)
- 3 (Some of the time) (3)
- 4 (4)
- 5 (Often) (5)

Q56 Isolated from others

- 1 (Hardly ever) (1)
- 2 (2)
- 3 (Some of the time) (3)
- 4 (4)
- 5 (Often) (5)

Q57 Anxiety

- 1 (Hardly ever) (1)
- 2 (2)
- 3 (Some of the time) (3)
- 4 (4)
- 5 (Often) (5)

Motivations Why did you decide to watch the online concert? Please rate how important each of the following reasons were to you: I watched the concert because…

Q58 I like the artist

- 1 (Not at all important) (1)
- 2 (2)
- 3 (3)
- 4 (4)
- 5 (Very important) (5)

Q59 I thought it would relax me

- 1 (Not at all important) (1)
- 2 (2)
- 3 (3)
- 4 (4)
- 5 (Very important) (5)

Q60 I thought it would make me feel less lonely

- 1 (Not at all important) (1)
- 2 (2)
- 3 (3)
- 4 (4)
- 5 (Very important) (5)

Q61 It would give me something to do to occupy my time

- 1 (Not at all important) (1)
- 2 (2)
- 3 (3)
- 4 (4)
- 5 (Very important) (5)

Q62 I thought it would be enjoyable

- 1 (Not at all important) (1)
- 2 (2)
- 3 (3)
- 4 (4)
- 5 (Very important) (5)

Q63 I was bored

- 1 (Not at all important) (1)
- 2 (2)
- 3 (3)
- 4 (4)
- 5 (Very important) (5)

Q64 I wanted to do something nice with friends or family

- 1 (Not at all important) (1)
- 2 (2)
- 3 (3)
- 4 (4)
- 5 (Very important) (5)

Q65 I wanted to feel more connected to other people

- 1 (Not at all important) (1)
- 2 (2)
- 3 (3)
- 4 (4)
- 5 (Very important) (5)

Q66 I thought it would provide a distraction

- 1 (Not at all important) (1)
- 2 (2)
- 3 (3)
- 4 (4)
- 5 (Very important) (5)

Q67 I thought it would cheer me up

- 1 (Not at all important) (1)
- 2 (2)
- 3 (3)
- 4 (4)
- 5 (Very important) (5)

Q68 I wanted to discover new music

- 1 (Not at all important) (1)
- 2 (2)
- 3 (3)
- 4 (4)
- 5 (Very important) (5)

Q121 I know the artist personally

- 1 (Not at all important) (1)
- 2 (2)
- 3 (3)
- 4 (4)
- 5 (Very important) (5)

Q69 Other reasons (please describe)

________________________________________________________________

Q70 To what extent did you engage in interactions surrounding the concert by reading, commenting, or donating?

- 1 (I did not read any comments) (1)
- 2 (2)
- 3 (3)
- 4 (4)
- 5 (I read many comments) (5)

Q71

- 1 (I did not speak about the concert with anyone) (1)
- 2 (2)
- 3 (3)
- 4 (4)
- 5 (I spoke about the concert at length or with several people) (5)

Q72 You may select multiple options for the responses below

- I did not share the concert online (1)
- I shared the concert on a social media platform (2)
- I shared it directly with family/friends (3)

Q73  You may select multiple options for the responses below

- I did not comment at all (1)
- I clicked on a reaction button (e.g. like (2)
- I wrote a brief comment (3)
- I wrote multiple brief comments (4)
- I wrote one or more detailed comments (5)

Q119 You may select multiple options for the responses below

- The performer asked for donations for themselves (1)
- The performer encouraged donations to a charity organization (2)
- The performer did not ask for any donations (6)
- Other (7) ________________________________________________

Q120 You may select multiple options for the responses below

- I donated to the performer (1)
- I donated to a charity that was recommended by the performer (2)
- I purchased a ticket for the event (3)
- I did not donate (4)
- Other way you contributed (5) ________________________________________________

Rate Rate your level of agreement with the statements below.

Q74 My experience in the streamed concert seemed similar to my experiences in a real concert.

- Completely disagree (1)
- Disagree (2)
- Neither disagree nor agree (3)
- Agree (4)
- Strongly agree (5)

Q75 While I was at the streamed concert, I had a sense of “being there” with the performers and audience members.

- Completely disagree (1)
- Disagree (2)
- Neither disagree nor agree (3)
- Agree (4)
- Strongly agree (5)

Q76 I was completely captivated by the streamed concert.

- Completely disagree (1)
- Disagree (2)
- Neither disagree nor agree (3)
- Agree (4)
- Strongly agree (5)

Q77 I felt like I was in the presence of other people who were online in the streamed concert.

- Completely disagree (1)
- Disagree (2)
- Neither disagree nor agree (3)
- Agree (4)
- Strongly agree (5)

Q78 I felt that the other people in the streamed concert were aware of my presence.

- Completely disagree (1)
- Disagree (2)
- Neither disagree nor agree (3)
- Agree (4)
- Strongly agree (5)

Q79 The people in the streamed concert appeared to be engaged and active to me.

- Completely disagree (1)
- Disagree (2)
- Neither disagree nor agree (3)
- Agree (4)
- Strongly agree (5)

Q80 During the streamed concert there were times where the computer interface seemed to disappear, and I felt like I was actually at the concert with the audience and performers.

- Completely disagree (1)
- Disagree (2)
- Neither disagree nor agree (3)
- Agree (4)
- Strongly agree (5)

Q81 In your opinion, was the concert good?

- 1 (Not at all) (1)
- 2 (2)
- 3 (3)
- 4 (4)
- 5 (Extremely) (5)

Q82 What was the size of the audience (e.g. 10, 100, 1000, or unsure)?

________________________________________________________________

Q83 How was the quality of the audio?

- 1 (Very poor) (1)
- 2 (2)
- 3 (3)
- 4 (4)
- 5 (Excellent) (5)

Q84 How was the quality of the video?

- 1 (Very poor) (1)
- 2 (2)
- 3 (3)
- 4 (4)
- 5 (Excellent) (5)

End of Block: Block 2

Start of Block: Block 3

Q85 Do you practice music (play an instrument/sing, sing in a choir, play in a band)?

- Yes (1)
- No (2)

Q86 How many months of musical training have you received?

________________________________________________________________

Q87 On average, how many hours per day do you actually spend listening to music, either while doing something else or as your main activity?

- 0 (1)
- 1-2 (2)
- 3-4 (3)
- 5-8 (4)
- 9 or more (5)

Q88 How important has music been in your life in the last 3 years?

- 1 (Not at all) (1)
- 2 (2)
- 3 (3)
- 4 (4)
- 5 (Extremely) (5)

Q89 What is your usual level of attention or involvement when you listen to music?

- 1 (Background only) (1)
- 2 (2)
- 3 (3)
- 4 (4)
- 5 (Total concentration) (5)

Empathy Please indicate the extent that each of the following statement describes you:

Q90 When I see someone being taken advantage of, I feel kind of protective toward them.

- 1 (Does not describe me well) (1)
- 2 (2)
- 3 (3)
- 4 (4)
- 5 (Describes me very well) (5)

Q91 When I see someone being treated unfairly, I sometimes don’t feel very much pity for them.

- 1 (Does not describe me well) (1)
- 2 (2)
- 3 (3)
- 4 (4)
- 5 (Describes me very well) (5)

Q92 I often have tender, concerned feelings for people less fortunate than me.

- 1 (Does not describe me well) (1)
- 2 (2)
- 3 (3)
- 4 (4)
- 5 (Describes me very well) (5)

Q93 I would describe myself as a pretty soft‐hearted person.

- 1 (Does not describe me well) (1)
- 2 (2)
- 3 (3)
- 4 (4)
- 5 (Describes me very well) (5)

Q94 Sometimes I don’t feel sorry for other people when they are having problems.

- 1 (Does not describe me well) (1)
- 2 (2)
- 3 (3)
- 4 (4)
- 5 (Describes me very well) (5)

Q95 Other people’s misfortunes do not usually disturb me a great deal.

- 1 (Does not describe me well) (1)
- 2 (2)
- 3 (3)
- 4 (4)
- 5 (Describes me very well) (5)

Q96 I am often quite touched by things that I see happen.

- 1 (Does not describe me well) (1)
- 2 (2)
- 3 (3)
- 4 (4)
- 5 (Describes me very well) (5)

Q118 Do you have any final comment for the experimenters?

________________________________________________________________

End of Block: Block 3

Start of Block: Link_to_raffle

Q122 Thank you taking the time to complete the survey!

 If you wish to take part in the raffle of 20 gift vouchers to Amazon or Apple App Store & iTunes, you can leave your contact details (email address) in a separate survey.

 Your contact details cannot be linked to your answers in this survey, and your email address will not be used for any other purpose than contacting you in case you happen to win one of the gift vouchers.

 Thank you once again for your effort!

End of Block: Link_to_raffle

## B) Norwegian, German, and Spanish

Streamed concerts study

Start of Block: Consent_block

Consent **Studietittel: Nettbaserte konsertopplevelser under tilstand av fysisk distansering** **Samtykkeskjema**

 **Bakgrunn og hensikt** Dette er en invitasjon til deltagelse i en forskningsstudie om nettbaserte konsertopplevelser i en tilværelse av fysisk distansering. Vi er interesserte i dine opplevelser fra den siste nettkonserten du opplevde. Denne studien er utført av en forskningsgruppe ved Universitetet i Oslo. Du kan kontakte Dana Swarbrick, dana.swarbrick@imv.uio.no om du har spørsmål eller kommentarer.   **Hva innebærer studien?** I løpet av studien vil du bli bedt om å svare på flere spørsmål vedrørende den siste nettkonserten du opplevde. Du vil bli spurt om din begeistring for musikken og dine inntrykk av situasjonen. Studien tar omtrent 10 til 15 minutter å gjennomføre. Mot slutten vil du kunne delta i et lotteri der du kan vinne 1 av 20 kuponger (200 NOK verdi). Informasjonen du oppgir med hensyn til lotteriet vil ikke kunne knyttes opp mot svarene du oppgir i spørreskjemaet.
   **Potensielle fordeler og ulemper** Deltakelse i denne studien skal ikke resultere i noen uheldige utfall.   **Hva vil skje med informasjonen om deg?** Dataen som er registrert om deg vil kun bli brukt i samsvar med hensiktene til studien beskrevet over. All data vil bli behandlet uten navn, personnummer eller annen gjenkjennelig informasjon. Det vil heller ikke være mulig å identifisere deg når resultatene av studien blir publisert.   **Frivillig deltakelse** Deltakelse i studien er frivillig. Du må være over 18 år for å delta. Du kan trekke tilbake samtykket ditt når som helst uten å oppgi en spesifikk grunn. Dette vil ikke ha noen konsekvenser for videre behandling. Om du ønsker å delta så vennligst bekreft ditt samtykke under før du fortsetter.    **Personvern** Informasjon som blir lagret om deg er kun svarene du oppgir i spørreskjemaet. Ingen identifiserende informasjon, slik som IP-adresse, vil bli lagret.    **Videreføring av data til tredjeparter** Svarene du oppgir vil bli kombinert med svarene til andre deltakere og lagret i en stor database. Informasjonen du oppgir vil ikke kunne assosieres med deg. Databasen vil bli åpnet for forskningsmiljøet etter at studien er ferdig, noe som er normal forskningspraksis.   **Finansiering og Universitetet i Oslo sin rolle** Studien er finansiert av et forskningsstipend fra Psykologisk Institutt og Det Humanistiske Fakultet ved Universitetet i Oslo.    **Informasjon om resultatene av studien** Du har krav på å motta informasjon om resultatene av studien. Om du ønsker å motta informasjon om resultatene til eksperimentet etter at studien er fullført, vennligst kontakt forskningsgruppen.   **Jeg har lest og er enig i betingelsene over**

- Ja (1)

Consent **Studientitle: Onlinekonzerterlebnisse unter Bedingungen von Kontaktvermeidung**     **Einverständniserklärung**   **Hintergrund und Ziel** Hiermit bitten wie Sie um die Teilnahme an einer Studie über Onlinekonzerterlebnisse unter Bedingungen von Kontaktvermeidung. Wir interessieren uns für Ihre Erlebnisse während Ihres letzten Onlinekonzerts. Diese Studie wird von einem Team an der Universität Oslo durchgeführt. Für Fragen und Anmerkungen können Sie Dana Swarbrick kontaktieren: dana.swarbrick@imv.uio.no   **Was bringt die Studie mit sich?** Während der Studie werden Sie gebeten, eine Zahl von Fragen bezüglich Ihres letzten Onlinekonzerts zu beantworten. Wir werden Sie fragen, ob Sie die Musik genossen haben und was Ihre Eindrücke der Situation waren. Die Teilnahme an der Studie benötigt in etwa 10 bis 15 Minuten. Am Ende können Sie an einer Verlosung teilnehmen, in der Sie einen von 20 Gutscheinen gewinnen können (im Wert von 20 Euro). Persönliche Daten, die Sie im Rahmen der Verlosung abgeben, können nicht mit Ihren Antworten in der Studie in Verbindung gebracht werden.     **Potentielle Vor- und Nachteile** Durch die Teilnahme an der Studie entstehen für Sie keine uns bekannten Nachteile.   **Was geschieht mit den Informationen über Sie?** Die Daten, die wir über Sie erhalten, werden nur im Rahmen des Studienziels wie oben beschrieben verwendet. Alle Daten werden ohne Namen, ID-Nummer oder andere Informationen verarbeitet, die direkt mit Ihnen in Verbindung gebracht werden können. Wenn die Studienergebnisse veröffentlicht werden, wird es nicht möglich sein, Sie aus den Ergebnissen zu identifizieren.   **Freiwilligkeit der Teilnahme** Die Teilnahme an der Studie ist freiwillig. Um teilnehmen zu können, müssen Sie mindestens 18 Jahre alt sein. Sie können Ihre Zustimmung jederzeit und ohne Angabe von Gründen widerrufen. Dies wird keine Konsequenzen für Sie haben. Wenn Sie gerne an der Studie teilnehmen möchten, stimmen Sie der untenstehenden Aussage zu, bevor Sie fortfahren.   **Privatsphäre** Über Sie gespeicherte Informationen sind lediglich die Antworten, die Sie im Rahmen des Fragebogens geben. Keine identifizierbaren Informationen, wie beispielsweise IP-Adresse, werden gespeichert.    **Freigabe von Material und Daten an Dritte** Ihre Antworten werden mit denen anderer Teilnehmer in einer großen Datenbank zusammengeführt; Ihre Antworten können nicht zu Ihnen zurückverfolgt werden. Diese Datenbank wird nach der Vollendung des Projektes veröffentlicht, was als optimale Vorgehensweise in psychologischer Forschung empfohlen wird.   **Förderung und die Rolle der Universität Oslo**  Die Studie wird durch Forschungsstipendien des Psychologieinstituts and des Instituts für Geisteswissenschaften der Universität Oslo, Norwegen, finanziert.   **Informationen über die Studienergebnisse**  Sie haben das Recht, Informationen über die Studienergebnisse zu erhalten. Bitte kontaktieren Sie dafür das Forschungsteam.     Ich habe die oben aufgeführten Bedingungen gelesen und akzeptiert

- Ja (1)

Consent Título del estudio: Experiencias de conciertos en línea bajo condiciones de distanciamiento físico   (Nota: usted puede seleccionar versiones en inglés, alemán o noruego de la encuesta desde el menú en la esquina superior derecha)
   **Consentimiento informado** **Antecedentes y objetivo** Esta es una invitación a participar en un estudio de investigación sobre experiencias de conciertos en línea en condiciones de distanciamiento físico. Estamos interesado(a)s ​​en sus experiencias durante el último concierto en línea que experimentó. Esta investigación está siendo realizada por un equipo de la Universidad de Oslo. Usted puede contactar a Dana Swarbrick, dana.swarbrick@imv.uio.no, para realizar cualquier pregunta y/o comentario.   **¿Qué implica el estudio?** Durante el estudio, se le solicitará que responda  varias preguntas con respecto al último concierto en línea que experimentó. Le preguntaremos sobre su experiencia de disfrute de la música y sus impresiones acerca de la situación. El estudio dura entre 10 y 15 minutos en completarse. Al final, podrá participar en una lotería en la que podrá ganar 1 de cada 20 cupones (valor de 20 USD). La información proporcionada con respecto a la lotería no se puede vincular a sus respuestas actuales.   **Posibles ventajas y desventajas** El estudio no incluye desventajas potenciales conocidas. **¿Qué pasará con la información sobre usted?** Las muestras y los datos que se registran sobre usted solo se usarán de acuerdo con el propósito del estudio como se describe anteriormente. Todos los datos y muestras se procesarán sin nombre, número de identificación u otro tipo de información directamente reconocible. No será posible identificarlo(a) en los resultados del estudio cuando se publiquen.   **Participación voluntaria** La participación en el estudio es voluntaria. Para participar debe ser mayor de 18 años.  Podrá abandonar la investigación en cualquier momento, sin indicar ningún motivo en particular. Esto no tendrá ninguna consecuencia para su tratamiento posterior. Si desea participar, confirme su consentimiento a continuación antes de continuar.   **Privacidad** La información que se conserva  sobre usted son solo las respuestas que da en el cuestionario. No se guarda ninguna información que lo identifique, como la dirección IP.   **Liberación de material y datos a terceros.** Sus respuestas se juntan con las respuestas de lo(a)s otro(a)s participantes en una gran base de datos, sus respuestas no  pueden ser rastreadas hasta usted. Esta base de datos se publicará en un repositorio abierto al finalizar el proyecto, lo que se recomienda como mejor práctica en cualquier investigación psicológica.   **Financiamiento  y el rol de la Universidad de Oslo.** El estudio es financiado por becas de investigación del Departamento de Psicología y la Facultad de Humanidades de la Universidad de Oslo, Noruega.   **Información sobre el resultado del estudio.** Usted tiene derecho a recibir información sobre los resultados del estudio.  Para hacerlo puede ponerse en contacto con el equipo de investigación.   **He leído y acepto los términos anteriores**

- Sí (1)

End of Block: Consent_block

Start of Block: Block 1

Information Velkommen til **«Nettbaserte konsertopplevelser under fysisk distansering»** studien.   Vi ønsker å forstå hva som bidrar til opplevelsene av konserter på nett. Dette spørreskjema vil ta omtrent 15-20 minutter å gjennomføre.

Information
Wilkommen zur **“Onlinestudie zu Konzerterlebnissen unter den Bedingungen des social distancing“**
 
Unser Ziel ist es zu verstehen, was zu den Erlebnissen eines Onlinekonzertes beitragen kann. Das Ausfüllen der folgenden Umfrage benötigt etwa 10-15 Minuten.

Information Bienvenido(a) al estudio "**Experiencias de conciertos en línea bajo condiciones de distanciamiento físico**".   Nuestro objetivo es comprender qué contribuye a la experiencia de un concierto en línea. La siguiente encuesta debería tomarle aproximadamente 10-15 minutos.  

Q1 Har du nylig sett en nettkonsert i mer enn 15 minutter?

- Ja (1)
- Nei (2)

Q1 Haben Sie in letzter Zeit ein Onlinekonzert mehr als 15 Minuten lang verfolgt?

- Ja (1)
- Nein (2)

Q1 ¿Ha visto recientemente algún concierto en línea por más de 15 minutos?

- Sí (1)
- No (2)

Display This Question:

If Have you recently watched an online concert for more than 15 minutes? = No

Q2 For at du skal kunne svare på disse spørsmålene, er det viktig at du har noe erfaring med nettkonserter. Vennligst bruk minst 15 minutter på å se en nettkonsert av ditt ønske.

Q2 Um die folgenden Fragen beantworten zu können ist es wichtig, dass Sie entsprechende Erfahrung mit Onlinekonzerten haben. Bitte nehmen Sie sich mindestens 15 Minuten Zeit, um ein Onlinekonzert Ihrer Wahl zu verfolgen, bevor Sie an der Studie teilnehmen.

Q2 Para que pueda responder estas preguntas, es importante que haya tenido una experiencia adecuada con los conciertos en línea. Dedique al menos 15 minutos a ver un concierto en línea de su elección antes de responder esta encuesta.

Skip To: End of Survey If In order for you to answer these questions it is important that you have adequate experience with... Is Displayed

age Hvor gammel er du?

________________________________________________________________

age Wie alt sind Sie?

________________________________________________________________

age ¿Cuantos años tiene?

________________________________________________________________

Display This Question:

If If How old are you?<o:p></o:p> Text Response Is Less Than 18

Not_old Vi beklager. Om du er under 18 år sier loven at du ikke kan gi eget samtykke.

Not_old Es tut uns leid, leider kannst Du im Alter unter 18 Jahren gemäß der Ethikgesetzte keine Einverständniserklärung abgeben.

Not_old Lo sentimos. Si tiene menos de 18 años, las leyes éticas establecen que no tiene la facultad de dar su consentimiento informado.

Skip To: End of Survey If We are sorry. If you are under 18 years old, ethical laws state that you do not have the ability... Is Displayed

gender Hvilket kjønn er du?

- Kvinne (1)
- Mann (2)
- Foretrekker å ikke si (3)
- Foretrekker å beskrive selv (4) ________________________________________________

gender Was ist Ihr Geschlecht?

- Männlich (1)
- Weiblich (2)
- Keine Angabe (3)
- Eigene Beschreibung (4) ________________________________________________

gender ¿Cuál es su género?

- Mujer (1)
- Hombre (2)
- Prefiero no decirlo (3)
- Prefiero describirlo yo (4) ________________________________________________

country Hvilket land bor du i?

________________________________________________________________

country In welchem Land wohnen Sie?

________________________________________________________________

country ¿Cuál es su país de residencia?

________________________________________________________________

End of Block: Block 1

Start of Block: Block 2

Q6 Hvor ofte drar du på konsert vanligvis (før distanseringstiltakene)?

- Aldri (1)
- Mindre enn én gang i måneden (2)
- En eller to ganger i måneden (3)
- Omtrent hver uke eller annenhver uke (4)
- Mer enn en gang i uken (5)

Q6
 Wie häufig besuchen Sie normalerweise Livekonzerte (ohne Maßnahmen zur Kontaktvermeidung)?

- Nie (1)
- Seltener als einmal im Monat (2)
- Ein- bis zweimal im Monat (3)
- Circa jede bis jede zweite Woche (4)
- Mehr als einmal die Woche (5)

Q6 Usualmente ¿con qué frecuencia asiste a conciertos musicales en vivo (sin medidas de distanciamiento social)?

- Nunca (1)
- Menos de una vez al mes (2)
- Una o dos veces al mes (3)
- Aproximadamente cada semana o cada dos semanas (4)
- Más de una vez a la semana (5)

Q7 Hvor ofte har du sett på videoopptak av konserter den siste måneden?

- Aldri (1)
- Mindre enn én gang i måneden (2)
- En eller to ganger i måneden (3)
- Omtrent hver uke eller annenhver uke (4)
- Mer enn en gang i uken (5)

Q7 Wie häufig haben Sie Aufnahmen von Konzerten innerhalb des vergangenen Monats angesehen?

- Nie (1)
- Seltener als einmal im Monat (2)
- Ein- bis zweimal im Monat (3)
- Circa jede bis jede zweite Woche (4)
- Mehr als einmal die Woche (5)

Q7 ¿Con qué frecuencia ha visto conciertos grabados en video en el último mes?

- Nunca (1)
- Menos de una vez al mes (2)
- Una o dos veces al mes (3)
- Aproximadamente cada semana o cada dos semanas (4)
- Más de una vez a la semana (5)

Q8 Hvor ofte har du sett på live-stream konserter den siste måneden?

- Aldri (1)
- Mindre enn én gang i måneden (2)
- En eller to ganger i måneden (3)
- Omtrent hver uke eller annenhver uke (4)
- Mer enn en gang i uken (5)

Q8 Wie häufig haben Sie live übertragene Konzerte (live-streamed) innerhalb des vergangenen Monats angesehen?

- Nie (1)
- Seltener als einmal im Monat (2)
- Ein- bis zweimal im Monat (3)
- Circa jede bis jede zweite Woche (4)
- Mehr als einmal die Woche (5)

Q8 ¿Con qué frecuencia ha visto conciertos transmitidos en vivo en el último mes?

- Nunca (1)
- Menos de una vez al mes (2)
- Una o dos veces al mes (3)
- Aproximadamente cada semana o cada dos semanas (4)
- Más de una vez a la semana (5)

| Page Break |  |
| --- | --- |

Copy_url Vennligst kopier og lim inn lenken/URL fra den siste konserten du så eller hørte på nett i minst 15 minutter.

________________________________________________________________

Copy_url Bitte kopieren Sie den Link/die URL des letzten Onlinekonzerts, das Sie für mindestens 15 Minuten angehört oder -gesehen haben, und fügen Sie den Link/die URL hier ein:

________________________________________________________________

Copy_url Por favor copie y pegue el enlace / URL de donde accedió al último concierto que escuchó o vio en línea durante al menos 15 minutos:

________________________________________________________________

No_url

- Jeg har ikke lenken/URL (1)

No_url

- Ich habe keinen Link/keine URL (1)

No_url

- No tengo un enlace/URL (1)

Display This Question:

If = I don't have a link/URL

Q9 Vennligst oppgi plattformen du brukte for å se konserten (f.eks. Facebook, YouTube)

________________________________________________________________

Q9 Bitte tragen Sie ein, auf welcher Plattform Sie das Konzert angesehen haben (z.B. Facebook, YouTube).

________________________________________________________________

Q9 Por favor indique la plataforma en la que vio el concierto. (Ej. Facebook, YouTube)

________________________________________________________________

Q10 Hvor mye av konserten så du på (i minutter)?

________________________________________________________________

Q10 Wie lange (in Minuten) haben Sie das Konzert verfolgt?

________________________________________________________________

Q10 ¿Cuántos minutos vio del concierto?

________________________________________________________________

Q11 Var konserten strømmet live eller spilt inn på forhånd?

- Jeg så på en live strømmekonsert (mens konserten pågikk) (1)
- Jeg så på en strømmekonsert, men ikke live (etter konserten var ferdig) (2)
- Jeg så på en konsert som var spilt inn på forhånd (3)
- Usikker (4)

Q11 Welche Art Konzert haben Sie gesehen?

- Ich habe ein live übertragenes (live-streamed) Konzert in Echtzeit gesehen (während es stattfand) (1)
- Ich habe ein live übertragenes (live-streamed) Konzert gesehen, jedoch nicht in Echtzeit (nachdem das Konzert bereits beendet war) (2)
- Ich habe eine Aufnahme eines Konzerts gesehen (3)
- Nicht sicher (4)

Q11 ¿Qué tipo de concierto vio?

- Vi un concierto en vivo en tiempo real (como sucedió) (1)
- Vi un concierto en vivo, no en tiempo real (después de que el concierto había terminado) (2)
- Vi un concierto pregrabado (3)
- o No estoy seguro(a) (4)

Q13 Hva var navnet på artisten/bandet/gruppen? (f.eks. Cher, The Who, Berlin Philharmonics)

________________________________________________________________

Q13 Wie lautet der/die Name(n) des/der Künstler(s)? (z.B. Cher, The Who, Berliner Philharmoniker)

________________________________________________________________

Q13 Cuál es el nombre del artista o de los artistas? (e.g. Cher, The Who, Orquesta Filarmónica de Berlin)

________________________________________________________________

Q14 Hvilken sjanger var konserten?

________________________________________________________________

Q14 Welchem Genre gehörte das Konzert an?

________________________________________________________________

Q14 ¿De qué género musical fue el concierto?

________________________________________________________________

Q15 I hvilke omgivelser var konserten? (f.eks. kjøkken, konserthall)

________________________________________________________________

Q15 Was war der Ort/die Umgebung des Konzerts? (z.B. Küche, Club, Konzerthalle)

________________________________________________________________

Q15 ¿Cuál fue la locación/escenario del concierto? (Ej. cocina, sala de concierto)

________________________________________________________________

Q16 I hvilket land ble konserten spilt?

________________________________________________________________

Q16 In welchem Land und evtl. welcher Stadt fand das Konzert statt?

________________________________________________________________

Q16 ¿En qué país fue el concierto?

________________________________________________________________

Q17 Er du fan av artisten?

- 1 (Ikke fan i det hele tatt) (1)
- 2 (2)
- 3 (3)
- 4 (4)
- 5 (Stor fan) (5)

Q17 Sind Sie ein Fan des/der Künstler*in?

- 1 (Überhaupt nicht) (1)
- 2 (2)
- 3 (3)
- 4 (4)
- 5 (Sehr) (5)

Q17 ¿Es usted un(a) fan o fanático(a) del artista?

- 1 (Para nada) (1)
- 2 (2)
- 3 (3)
- 4 (4)
- 5 (Totalmente) (5)

Q18 Hvor viktig for deg var musikken som artisten fremførte? (f.eks. om en av sangene deres var viktig i en spesiell del av livet ditt)

- 1 (Ikke viktig i det hele tatt) (1)
- 2 (2)
- 3 (3)
- 4 (4)
- 5 (Ekstremt viktig) (5)

Q18 Wie wichtig ist die Musik dieses Künstlers/dieser Künstlerin für Sie? (z.B. eines der Lieder war in einer bedeutenden Zeit Ihres Lebens wichtig für Sie)

- 1 (Überhaupt nicht) (1)
- 2 (2)
- 3 (3)
- 4 (4)
- 5 (Sehr) (5)

Q18 ¿Qué tan importante es la música interpretada por este artista para usted? (por ejemplo, una de sus canciones fue importante durante un momento significativo de su vida)

- 1 (Para nada) (1)
- 2 (2)
- 3 (3)
- 4 (4)
- 5 (Extremadamente) (5)

Q19 Vennligst velg utstyret du brukte for å høre og se på konserten

Q19 Bitte wählen Sie das Gerät, mit dem Sie das Konzert gehört und gesehen haben, mithilfe der folgenden Fragen aus

Q19 Por favor seleccione el aparato o equipo que utilizó para escuchar y ver el concierto en las dos preguntas a continuación:

Q20 Vennligst oppgi hva slags skjerm du brukte for å se konserten på:

- Mobilskjerm (1)
- Nettbrett (2)
- Laptop (3)
- Dataskjerm (4)
- TV-skjerm (5)
- Projektor (6)
- Annet: Vennligst beskriv (7) ________________________________________________

Q20 Bitte wählen Sie den Bildschirm aus, auf dem Sie das Konzert gesehen haben:

- Handybildschirm (1)
- Tablet (2)
- Laptop (3)
- Desktopbildschirm (4)
- großer Fernsehbildschirm (5)
- große Projektion (6)
- Andere: bitte erläutern (7) ________________________________________________

Q20 Por favor marque la pantalla en la que vio el concierto:

- Pantalla de celular (1)
- Tablet (2)
- Laptop o Notebook (3)
- Pantalla de escritorio (4)
- Una pantalla de televisor grande (5)
- Una proyección en una gran pantalla (6)
- Otro: Por favor describa (7) ________________________________________________

Q21 Vennligst indiker hva slags lydutstyr du brukte:

- Enkle hodetelefoner/øreplugger (1)
- Hodetelefoner/øreplugger av høy kvalitet (2)
- Innebygde høyttalere (3)
- Eksterne høyttalere (4)
- Høyttalere av høy kvalitet (5)
- Annet: Vennligst beskriv (6) ________________________________________________

Q21 Bitte wählen Sie das verwendete Audiogerät aus:

- Einfache Kopfhörer (1)
- Hochwertige Kopfhörer (2)
- Kleine Lautsprecher (3)
- Große Lautsprecher (4)
- Hochwertige Lautsprecher (5)
- Andere: bitte erläutern (6) ________________________________________________

Q21 Por favor marque el equipo de audio que utilizó:

- Audífonos/auriculares simples (1)
- Audífonos/auriculares de alta calidad (2)
- Altavoces/parlantes incorporados (3)
- Altavoces/parlantes externos (4)
- Altavoces/parlantes de alta calidad (5)
- Otro: Por favor describa (6) ________________________________________________

Q22 Hvilket oppmerksomhetsnivå hadde du under konserten?

- 1 (Kun bakgrunn) (1)
- 2 (2)
- 3 (3)
- 4 (4)
- 5 (Total konsentrasjon) (5)

Q22 Was war Ihr Aufmerksamkeitsniveau, während Sie das Konzert verfolgt haben?

- 1 (Lediglich Hintergrund) (1)
- 2 (2)
- 3 (3)
- 4 (4)
- 5 (Totale Konzentration) (5)

Q22 ¿Cuál fue su nivel de atención cuando estaba viendo el concierto?

- 1 (Solo música de fondo/ambiental) (1)
- 2 (2)
- 3 (3)
- 4 (4)
- 5 (Concentración total) (5)

| Page Break |  |
| --- | --- |

Q23 Hvor ofte interagerte musikerne med hverandre?

- Det var bare en musikker (1)
- 1 (Ikke ofte) (2)
- 2 (3)
- 3 (4)
- 4 (5)
- 5 (Veldig ofte) (6)

Q23 Wie häufig interagierten die Künstler*innen miteinander?

- Es gab nur eine*n Künstler*in (1)
- 1 (Nicht häufig) (2)
- 2 (3)
- 3 (4)
- 4 (5)
- 5 (Sehr häufig) (6)

Q23 ¿Qué tan frecuentemente lo(a)s intérpretes interactuaban entre ello(a)s?

- Solo había un(a) intérprete (1)
- 1 (No tan frecuentemente) (2)
- 2 (3)
- 3 (4)
- 4 (5)
- 5 (Muy frecuentemente) (6)

Q24 Hvor ofte interagerte musikerne med publikum?

- Aldri (1)
- Én eller to ganger (2)
- Av og til (3)
- Jevnlig (4)
- Veldig ofte (5)

Q24 Wie häufig interagierten die Künstler*innen mit dem Publikum?

- Nie (1)
- Ein- oder zweimal (2)
- Gelegentlich (3)
- Oft (4)
- Sehr häufig (5)

Q24 ¿Qué tan frecuentemente lo(a)s intérpretes interactuaban con la audiencia?

- Nunca (1)
- Una o dos veces (2)
- Ocasionalmente (3)
- Frecuentemente (4)
- Muy frecuentemente (5)

Q25 Hvor mye merket du tilstedeværelsen til de andre publikummerne?

- 1 (Ikke i det hele tatt) (1)
- 2 (2)
- 3 (3)
- 4 (4)
- 5 (Veldig mye) (5)

Q25 Wie sehr waren Sie sich der Anwesenheit anderer Menschen im Publikum bewusst?

- 1 (Überhaupt nicht) (1)
- 2 (2)
- 3 (3)
- 4 (4)
- 5 (Sehr) (5)

Q25 ¿Cuánto notó la presencia de otras personas de la audiencia?

- 1 (Nada) (1)
- 2 (2)
- 3 (3)
- 4 (4)
- 5 (Mucho) (5)

Q26 Kjente du noen av de andre publikummerne?

- Ja: Vennligst indiker omtrent hvor mange du kjente: (1) ________________________________________________
- Nei (2)

Q26 Kannten Sie andere Menschen, die sich ebenfalls im Publikum befanden?

- Ja (bitte geben Sie an, wie viele Leute Sie in etwa aus dem Publikum kannten) (1) ________________________________________________
- Nein (2)

Q26 ¿Conocías a otras personas de las que estaban en la audiencia?

- Sí (indique aproximadamente a cuántas personas conocía en la audiencia) (1) ________________________________________________
- No (2)

Q27 Hvor fremtredende var temaet om sosial distansering som følge av coronaviruset (f.eks diskuterte musikerne eller publikummet temaet eller ble det oppfordret til donasjoner)?

- 1 (Ikke fremtredende i det hele tatt) (1)
- 2 (2)
- 3 (3)
- 4 (4)
- 5 (Veldig fremtredende) (5)

Q27 Wie sehr wurden die durch das Coronavirus verursachten Umstände der Kontaktvermeidung vergegenwärtigt (z.B. diskutierten die Künstler*innen oder Leute im Publikum über das Thema oder ermunterten zum Spenden)?

- 1 (Überhaupt nicht vergegenwärtigt) (1)
- 2 (2)
- 3 (3)
- 4 (4)
- 5 (Sehr vergegenwärtigt) (5)

Q27 ¿Qué tan destacadas fueron las circunstancias de distanciamiento social inducidas por el coronavirus (por ejemplo, el/la artista o los miembros de la audiencia discutieron el tema o motivaron las donaciones)? Qué importancia le dieron a las circunstancias de distanciamiento social….

- 1 (Nada) (1)
- 2 (2)
- 3 (3)
- 4 (4)
- 5 (Mucho) (5)

Q28 Vennligst beskriv med dine egne ord hvordan konserten fikk deg til å føle deg:

________________________________________________________________

Q28 Bitte beschreiben Sie in eigenen Worten, wie Sie sich während des Konzerts gefühlt haben:

________________________________________________________________

Q28 Por favor, describa con sus propias palabras como lo(a) hizo sentir ver el video:

________________________________________________________________

Q29 Hvor knyttet følte du deg til musikeren/musikerne?

- 1 (Ikke i det hele tatt) (1)
- 2 (2)
- 3 (3)
- 4 (4)
- 5 (Ekstremt) (5)

Q29 Wie verbunden fühlten Sie sich mit dem/den Künstler(n)?

- 1 (Überhaupt nicht) (1)
- 2 (2)
- 3 (3)
- 4 (4)
- 5 (Sehr) (5)

Q29 ¿Qué tan conectado(a) se sintió con lo(a)s intérpretes?

- 1 (En absoluto) (1)
- 2 (2)
- 3 (3)
- 4 (4)
- 5 (Extremadamente) (5)

Q30 Hvor knyttet følte du deg til de andre publikummerne?

- 1 (Ikke i det hele tatt) (1)
- 2 (2)
- 3 (3)
- 4 (4)
- 5 (Ekstremt) (5)

Q30 Wie verbunden fühlten Sie sich mit anderen Menschen aus dem Publikum?

- 1 (Überhaupt nicht) (1)
- 2 (2)
- 3 (3)
- 4 (4)
- 5 (Sehr) (5)

Q30 ¿Qué tan conectado(a) se sintió con los otros miembros de la audiencia?

- 1 (En absoluto) (1)
- 2 (2)
- 3 (3)
- 4 (4)
- 5 (Extremadamente) (5)

Q31 Var det noen andre som så konserten med deg i samme fysiske rom?

- Ja (vennligst indiker hvor mange): (1) ________________________________________________
- Nei (2)

Q31 Sahen Sie das Konzert gemeinsam mit anderen Menschen im selben physischen Raum?

- Ja (bitte geben Sie an wie viele) (1) ________________________________________________
- Nein (2)

Q31 ¿Había otras personas viendo el concierto con usted en el mismo espacio físico?

- Sí (por favor indique cuántos) (1) ________________________________________________
- No (2)

Q32 I hvilken grad følte du at du delte følelser med andre publikummere?

- 1 (Ikke i det hele tatt) (1)
- 2 (2)
- 3 (3)
- 4 (4)
- 5 (I ekstremt stor grad) (5)

Q32 Wie sehr hatten Sie das Gefühl, mit anderen Menschen aus dem Publikum Emotionen zu teilen?

- 1 (Überhaupt nicht) (1)
- 2 (2)
- 3 (3)
- 4 (4)
- 5 (Sehr) (5)

Q32 ¿Hasta qué punto sintió que compartió emociones con otros miembros de la audiencia?

- 1 (En absoluto) (1)
- 2 (2)
- 3 (3)
- 4 (4)
- 5 (Extremadamente) (5)

| Page Break |  |
| --- | --- |

Feel Vennligst beskriv hvordan konserten fikk deg til å føle deg ved å bruke adjektivene under:

Feel Bitte beschreiben Sie mithilfe der folgenden Skala, wie Sie sich während des Konzerts gefühlt haben:

Feel Por favor describa cómo lo(a) hizo sentir el concierto:

Q33 Rørt

- 1 (Ikke i det hele tatt) (1)
- 2 (2)
- 3 (3)
- 4 (4)
- 5 (Ekstremt) (5)

Q33 Bewegt/berührt

- 1 (Überhaupt nicht) (1)
- 2 (2)
- 3 (3)
- 4 (4)
- 5 (Sehr) (5)

Q33 Emocionado(a) (conmovido(a)/tocado(a))

- 1 (En absoluto) (1)
- 2 (2)
- 3 (3)
- 4 (4)
- 5 (Extremadamente) (5)

Q34 Fredelig/avslappet

- 1 (Ikke i det hele tatt) (1)
- 2 (2)
- 3 (3)
- 4 (4)
- 5 (Ekstremt) (5)

Q34 Friedlich/entspannt

- 1 (Überhaupt nicht) (1)
- 2 (2)
- 3 (3)
- 4 (4)
- 5 (Sehr) (5)

Q34 Tranquilo(a)/relajado(a)

- 1 (En absoluto) (1)
- 2 (2)
- 3 (3)
- 4 (4)
- 5 (Extremadamente) (5)

Q35 Energisk/våken

- 1 (Ikke i det hele tatt) (1)
- 2 (2)
- 3 (3)
- 4 (4)
- 5 (Ekstremt) (5)

Q35 Energetisch/wach

- 1 (Überhaupt nicht) (1)
- 2 (2)
- 3 (3)
- 4 (4)
- 5 (Sehr) (5)

Q35 Energético(a)/despierto(a)

- 1 (En absoluto) (1)
- 2 (2)
- 3 (3)
- 4 (4)
- 5 (Extremadamente) (5)

Q36 Glad

- 1 (Ikke i det hele tatt) (1)
- 2 (2)
- 3 (3)
- 4 (4)
- 5 (Ekstremt) (5)

Q36 Glücklich/freudig

- 1 (Überhaupt nicht) (1)
- 2 (2)
- 3 (3)
- 4 (4)
- 5 (Sehr) (5)

Q36 Feliz/alegre

- 1 (En absoluto) (1)
- 2 (2)
- 3 (3)
- 4 (4)
- 5 (Extremadamente) (5)

Q37 Trist/melankolsk

- 1 (Ikke i det hele tatt) (1)
- 2 (2)
- 3 (3)
- 4 (4)
- 5 (Ekstremt) (5)

Q37 Traurig/melancholisch

- 1 (Überhaupt nicht) (1)
- 2 (2)
- 3 (3)
- 4 (4)
- 5 (Sehr) (5)

Q37 Triste/melancólico(a)

- 1 (En absoluto) (1)
- 2 (2)
- 3 (3)
- 4 (4)
- 5 (Extremadamente) (5)

Q38 Anspent/nervøs

- 1 (Ikke i det hele tatt) (1)
- 2 (2)
- 3 (3)
- 4 (4)
- 5 (Ekstremt) (5)

Q38 Angespannt/besorgt

- 1 (Überhaupt nicht) (1)
- 2 (2)
- 3 (3)
- 4 (4)
- 5 (Sehr) (5)

Q38 Tenso(a)/ansioso(a)

- 1 (En absoluto) (1)
- 2 (2)
- 3 (3)
- 4 (4)
- 5 (Extremadamente) (5)

Q39 Følsom/varm

- 1 (Ikke i det hele tatt) (1)
- 2 (2)
- 3 (3)
- 4 (4)
- 5 (Ekstremt) (5)

Q39 Zärtlich/warm

- 1 (Überhaupt nicht) (1)
- 2 (2)
- 3 (3)
- 4 (4)
- 5 (Sehr) (5)

Q39 Agradado(a)/cálido(a)

- 1 (En absoluto) (1)
- 2 (2)
- 3 (3)
- 4 (4)
- 5 (Extremadamente) (5)

KAMMUS-S Vennligst indikér hvorvidt og i hvor stor grad du opplevde de følgende fornemmelsene, følelsene eller handlingene under konserten:

KAMMUS-S Bitte geben Sie an, ob und in welchem Ausmaß Sie die folgenden Empfindungen, Gefühle oder Reaktionen während des Konzerts erlebt haben:

KAMMUS-S Por favor indique si experimentó alguna de las siguientes sensaciones, sentimientos o acciones durante el concierto y, de ser así, en qué medida:

Q40 Tårer

- 1 (Ikke i det hele tatt) (1)
- 2 (2)
- 3 (3)
- 4 (4)
- 5 (5)
- 6 (Veldig mye) (6)

Q40 Tränen

- 1 (Überhaupt nicht) (1)
- 2 (2)
- 3 (3)
- 4 (4)
- 5 (5)
- 6 (Äußerst) (6)

Q40 Lágrimas

- 1 (En absoluto) (1)
- 2 (2)
- 3 (3)
- 4 (4)
- 5 (5)
- 6 (Mucho) (6)

Q41 Gåsehud eller frysninger

- 1 (Ikke i det hele tatt) (1)
- 2 (2)
- 3 (3)
- 4 (4)
- 5 (5)
- 6 (Veldig mye) (6)

Q41 Gänsehaut oder Haare standen mir zu Berge

- 1 (Überhaupt nicht) (1)
- 2 (2)
- 3 (3)
- 4 (4)
- 5 (5)
- 6 (Äußerst) (6)

Q41 Escalofríos/Erizar la piel

- 1 (En absoluto) (1)
- 2 (2)
- 3 (3)
- 4 (4)
- 5 (5)
- 6 (Mucho) (6)

Q42 En varm følelse midt i brystet

- 1 (Ikke i det hele tatt) (1)
- 2 (2)
- 3 (3)
- 4 (4)
- 5 (5)
- 6 (Veldig mye) (6)

Q42 Ein warmes Gefühl im Inneren der Brust

- 1 (Überhaupt nicht) (1)
- 2 (2)
- 3 (3)
- 4 (4)
- 5 (5)
- 6 (Äußerst) (6)

Q42 Una sensación cálida en el centro del pecho

- 1 (En absoluto) (1)
- 2 (2)
- 3 (3)
- 4 (4)
- 5 (5)
- 6 (Mucho) (6)

Q43 Gråtkvalt

- 1 (Ikke i det hele tatt) (1)
- 2 (2)
- 3 (3)
- 4 (4)
- 5 (5)
- 6 (Veldig mye) (6)

Q43 Kloß im Hals

- 1 (Überhaupt nicht) (1)
- 2 (2)
- 3 (3)
- 4 (4)
- 5 (5)
- 6 (Äußerst) (6)

Q43 Un nudo en la garganta

- 1 (En absoluto) (1)
- 2 (2)
- 3 (3)
- 4 (4)
- 5 (5)
- 6 (Mucho) (6)

Q44 Forfrisket, energisk, eller oppkvikket

- 1 (Ikke i det hele tatt) (1)
- 2 (2)
- 3 (3)
- 4 (4)
- 5 (5)
- 6 (Veldig mye) (6)

Q44 Erfrischt, energiegeladen oder beschwingt

- 1 (Überhaupt nicht) (1)
- 2 (2)
- 3 (3)
- 4 (4)
- 5 (5)
- 6 (Äußerst) (6)

Q44 Refrescado(a), energizado(a) o regocijado(a)

- 1 (En absoluto) (1)
- 2 (2)
- 3 (3)
- 4 (4)
- 5 (5)
- 6 (Mucho) (6)

Q45 Ønske om å bevege kroppen, slik som tramping med foten, hodenikking, eller dansing

- 1 (Ikke i det hele tatt) (1)
- 2 (2)
- 3 (3)
- 4 (4)
- 5 (5)
- 6 (Veldig mye) (6)

Q45 Lust sich zu bewegen, z.B. Tanzen oder Tappen der Hände oder Füße

- 1 (Überhaupt nicht) (1)
- 2 (2)
- 3 (3)
- 4 (4)
- 5 (5)
- 6 (Äußerst) (6)

Q45 Con deseo de mover su cuerpo como golpear una superficie con los dedos de los pies, mover la cabeza o bailar

- 1 (En absoluto) (1)
- 2 (2)
- 3 (3)
- 4 (4)
- 5 (5)
- 6 (Mucho) (6)

Q46 Le høyt

- 1 (Ikke i det hele tatt) (1)
- 2 (2)
- 3 (3)
- 4 (4)
- 5 (5)
- 6 (Veldig mye) (6)

Q46 Lautes Lachen

- 1 (Überhaupt nicht) (1)
- 2 (2)
- 3 (3)
- 4 (4)
- 5 (5)
- 6 (Äußerst) (6)

Q46 Reír a carcajadas

- 1 (En absoluto) (1)
- 2 (2)
- 3 (3)
- 4 (4)
- 5 (5)
- 6 (Mucho) (6)

Q47 Avslappet pust

- 1 (Ikke i det hele tatt) (1)
- 2 (2)
- 3 (3)
- 4 (4)
- 5 (5)
- 6 (Veldig mye) (6)

Q47 Entspanntes Atmen

- 1 (Überhaupt nicht) (1)
- 2 (2)
- 3 (3)
- 4 (4)
- 5 (5)
- 6 (Äußerst) (6)

Q47 Respirar relajadamente

- 1 (En absoluto) (1)
- 2 (2)
- 3 (3)
- 4 (4)
- 5 (5)
- 6 (Mucho) (6)

Q48 Annet (vennligst beskriv):

________________________________________________________________

Q48 Anderes (bitte beschreiben)

________________________________________________________________

Q48 Otro (por favor describa)

________________________________________________________________

Stat Vennligst indikér hvorvidt og i hvor stor grad hver av de følgende utsagnene er sanne:

Stat Bitte gebenn Sie an, ob und in welchem Ausmaß die folgenden Aussagen auf Sie zutreffen:

Stat Por favor evalúe en qué medida cada una de las siguientes afirmaciones es verdadera:

Q49 Jeg følte en ekstraordinær følelse av å være velkommen.

- 1 (Ikke i det hele tatt) (1)
- 2 (2)
- 3 (3)
- 4 (4)
- 5 (5)
- 6 (Veldig mye) (6)

Q49 Ich fühlte mich in besonderer Weise willkommen oder nahm jemanden herzlich auf.

- 1 (Überhaupt nicht) (1)
- 2 (2)
- 3 (3)
- 4 (4)
- 5 (5)
- 6 (Äußerst) (6)

Q49 Sentí una extraordinaria sensación de bienvenida o de ser bienvenido(a).

- 1 (En absoluto) (1)
- 2 (2)
- 3 (3)
- 4 (4)
- 5 (5)
- 6 (Mucho) (6)

Q50 Jeg observerte en eksepsjonell følelse av nærhet oppstå.

- 1 (Ikke i det hele tatt) (1)
- 2 (2)
- 3 (3)
- 4 (4)
- 5 (5)
- 6 (Veldig mye) (6)

Q50 Ich wurde Zeuge davon, wie plötzlich eine große Nähe entstanden ist.

- 1 (Überhaupt nicht) (1)
- 2 (2)
- 3 (3)
- 4 (4)
- 5 (5)
- 6 (Äußerst) (6)

Q50 Observé que surgió una sensación de cercanía excepcional.

- 1 (En absoluto) (1)
- 2 (2)
- 3 (3)
- 4 (4)
- 5 (5)
- 6 (Mucho) (6)

Q51 Jeg opplevde positive følelser.

- 1 (Ikke i det hele tatt) (1)
- 2 (2)
- 3 (3)
- 4 (4)
- 5 (5)
- 6 (Veldig mye) (6)

Q51 Ich hatte positive Gefühle.

- 1 (Überhaupt nicht) (1)
- 2 (2)
- 3 (3)
- 4 (4)
- 5 (5)
- 6 (Äußerst) (6)

Q51 Tuve sentimientos positivos.

- 1 (En absoluto) (1)
- 2 (2)
- 3 (3)
- 4 (4)
- 5 (5)
- 6 (Mucho) (6)

Feel Vennligst indikér hvorvidt og i hvor stor grad du opplevde de følgende følelsene like etterpå:

Feel Bitte geben Sie an, ob und in welchem Ausmaß die folgenden Aussagen auf Sie zutreffen:

Feel Por favor indique si tuvo cada uno de los siguientes sentimientos justo después y, en caso afirmativo, en qué medida:

Q52 Jeg følte for å fortelle noen hvor mye jeg bryr meg om dem.

- 1 (Ikke i det hele tatt) (1)
- 2 (2)
- 3 (3)
- 4 (4)
- 5 (5)
- 6 (I veldig stor grad) (6)

Q52 Ich hatte das Bedürfnis jemandem mitzuteilen, wie viel er/sie mir bedeutet.

- 1 (Überhaupt nicht) (1)
- 2 (2)
- 3 (3)
- 4 (4)
- 5 (5)
- 6 (Äußerst) (6)

Q52 Me sentí como diciéndole a alguien cuanto me importa

- 1 (En absoluto) (1)
- 2 (2)
- 3 (3)
- 4 (4)
- 5 (5)
- 6 (Mucho) (6)

Q53 Jeg hadde lyst til å gi noen en klem.

- 1 (Ikke i det hele tatt) (1)
- 2 (2)
- 3 (3)
- 4 (4)
- 5 (5)
- 6 (I veldig stor grad) (6)

Q53 Ich wollte jemanden umarmen.

- 1 (Überhaupt nicht) (1)
- 2 (2)
- 3 (3)
- 4 (4)
- 5 (5)
- 6 (Äußerst) (6)

Q53 Me dieron ganas de abrazar a alguien.

- 1 (En absoluto) (1)
- 2 (2)
- 3 (3)
- 4 (4)
- 5 (5)
- 6 (Mucho) (6)

| Page Break |  |
| --- | --- |

Loneliness Siden begynnelsen av tiltakene om sosial distansering, hvor ofte har du følt på følgende:

Loneliness Wie oft haben Sie das Folgende seit Beginn der Maßnahmen zur Kontaktvermeidung gefühlt:

Loneliness Desde el inicio de las medidas de distanciamiento social, con qué frecuencia ha sentido lo siguiente:

Q54 Ensomhet

- 1 (Nesten aldri) (1)
- 2 (2)
- 3 (Noen ganger) (3)
- 4 (4)
- 5 (Ofte) (5)

Q54 Einsamkeit

- 1 (Kaum) (1)
- 2 (2)
- 3 (Manchmal) (3)
- 4 (4)
- 5 (Häufig) (5)

Q54 Soledad

- 1 (Casi nunca) (1)
- 2 (2)
- 3 (Algunas veces) (3)
- 4 (4)
- 5 (Frecuentemente) (5)

Q55 Mangel på samvær

- 1 (Nesten aldri) (1)
- 2 (2)
- 3 (Noen ganger) (3)
- 4 (4)
- 5 (Ofte) (5)

Q55 Fehlende Gesellschaft

- 1 (Kaum) (1)
- 2 (2)
- 3 (Manchmal) (3)
- 4 (4)
- 5 (Häufig) (5)

Q55 Falta de compañía

- 1 (Casi nunca) (1)
- 2 (2)
- 3 (Algunas veces) (3)
- 4 (4)
- 5 (Frecuentemente) (5)

Q56 Isolasjon fra andre

- 1 (Nesten aldri) (1)
- 2 (2)
- 3 (Noen ganger) (3)
- 4 (4)
- 5 (Ofte) (5)

Q56 Isolation von anderen

- 1 (Kaum) (1)
- 2 (2)
- 3 (Manchmal) (3)
- 4 (4)
- 5 (Häufig) (5)

Q56 Aislado(a) de los demás

- 1 (Casi nuca) (1)
- 2 (2)
- 3 (Algunas veces) (3)
- 4 (4)
- 5 (Frecuentemente) (5)

Q57 Angst

- 1 (Nesten aldri) (1)
- 2 (2)
- 3 (Noen ganger) (3)
- 4 (4)
- 5 (Ofte) (5)

Q57 Angst

- 1 (Kaum) (1)
- 2 (2)
- 3 (Manchmal) (3)
- 4 (4)
- 5 (Häufig) (5)

Q57 Ansiedad

- 1 (Casi nunca) (1)
- 2 (2)
- 3 (Algunas veces) (3)
- 4 (4)
- 5 (Frecuentemente) (5)

Motivations Hvorfor valgte du å se på nettkonserten? Vennligst vurder hvor viktig hver av de følgende grunnene var for deg. Jeg så på konserten fordi..

Motivations Warum haben Sie sich dazu entschieden, das Onlinekonzert zu sehen? Bitte wählen Sie aus, wie wichtig die folgenden Gründe jeweils für Sie waren: Ich habe das Konzert angesehen, weil…

Motivations ¿Por qué decidió ver el concierto en línea? Por favor evalúe la importancia de cada una de las siguientes razones para usted: vi el concierto porque...

Q58 Jeg liker artisten

- 1 (Ikke viktig i det hele tatt) (1)
- 2 (2)
- 3 (3)
- 4 (4)
- 5 (Veldig viktig) (5)

Q58 Ich den/die Künstler*in mag

- 1 (Überhaupt nicht wichtig) (1)
- 2 (2)
- 3 (3)
- 4 (4)
- 5 (Sehr wichtig) (5)

Q58 Me gusta el/la artista

- 1 (Nada importante) (1)
- 2 (2)
- 3 (3)
- 4 (4)
- 5 (Muy importante) (5)

Q59 Jeg tenkte det ville gjøre meg avslappet

- 1 (Ikke viktig i det hele tatt) (1)
- 2 (2)
- 3 (3)
- 4 (4)
- 5 (Veldig viktig) (5)

Q59 Ich dachte, dass es mich entspannen würde

- 1 (Überhaupt nicht wichtig) (1)
- 2 (2)
- 3 (3)
- 4 (4)
- 5 (Sehr wichtig) (5)

Q59 Pensé que me iba a relajar

- 1 (Nada importante) (1)
- 2 (2)
- 3 (3)
- 4 (4)
- 5 (Muy importante) (5)

Q60 Jeg tenkte det ville få meg til å føle meg mindre ensom

- 1 (Ikke viktig i det hele tatt) (1)
- 2 (2)
- 3 (3)
- 4 (4)
- 5 (Veldig viktig) (5)

Q60 Ich dachte, dass ich mich weniger einsam fühlen würde

- 1 (Überhaupt nicht wichtig) (1)
- 2 (2)
- 3 (3)
- 4 (4)
- 5 (Sehr wichtig) (5)

Q60 Pensé que me haría sentir menos solo(a)

- 1 (Nada importante) (1)
- 2 (2)
- 3 (3)
- 4 (4)
- 5 (Muy importante) (5)

Q61 Det ville gi meg noe å gjøre for og få tiden til å gå

- 1 (Ikke viktig i det hele tatt) (1)
- 2 (2)
- 3 (3)
- 4 (4)
- 5 (Veldig viktig) (5)

Q61 Es mich beschäftigen würde

- 1 (Überhaupt nicht wichtig) (1)
- 2 (2)
- 3 (3)
- 4 (4)
- 5 (Sehr wichtig) (5)

Q61 Me daría algo que hacer para ocupar mi tiempo

- 1 (Nada importante) (1)
- 2 (2)
- 3 (3)
- 4 (4)
- 5 (Muy importante) (5)

Q62 Jeg tenkte det ville være hyggelig

- 1 (Ikke viktig i det hele tatt) (1)
- 2 (2)
- 3 (3)
- 4 (4)
- 5 (Veldig viktig) (5)

Q62 Ich dachte, dass es unterhaltsam sein könnte

- 1 (Überhaupt nicht wichtig) (1)
- 2 (2)
- 3 (3)
- 4 (4)
- 5 (Sehr wichtig) (5)

Q62 Pensé que sería agradable

- 1 (Nada importante) (1)
- 2 (2)
- 3 (3)
- 4 (4)
- 5 (Muy importante) (5)

Q63 Jeg kjedet meg

- 1 (Ikke viktig i det hele tatt) (1)
- 2 (2)
- 3 (3)
- 4 (4)
- 5 (Veldig viktig) (5)

Q63 Mir langweilig war

- 1 (Überhaupt nicht wichtig) (1)
- 2 (2)
- 3 (3)
- 4 (4)
- 5 (Sehr wichtig) (5)

Q63 Estaba aburrido(a)

- 1 (Nada importante) (1)
- 2 (2)
- 3 (3)
- 4 (4)
- 5 (Muy importante) (5)

Q64 Jeg ville gjøre noe fint med venner eller familie

- 1 (Ikke viktig i det hele tatt) (1)
- 2 (2)
- 3 (3)
- 4 (4)
- 5 (Veldig viktig) (5)

Q64 Ich etwas Nettes mit Freunden oder Familie unternehmen wollte

- 1 (Überhaupt nicht wichtig) (1)
- 2 (2)
- 3 (3)
- 4 (4)
- 5 (Sehr wichtig) (5)

Q64 Quería hacer algo agradable con amigo(a)s o familiares

- 1 (Nada importante) (1)
- 2 (2)
- 3 (3)
- 4 (4)
- 5 (Muy importante) (5)

Q65 Jeg ville føle meg mer forent med andre mennesker

- 1 (Ikke viktig i det hele tatt) (1)
- 2 (2)
- 3 (3)
- 4 (4)
- 5 (Veldig viktig) (5)

Q65 Ich mich mehr mit anderen verbunden fühlen wollte

- 1 (Überhaupt nicht wichtig) (1)
- 2 (2)
- 3 (3)
- 4 (4)
- 5 (Sehr wichtig) (5)

Q65 Quería sentirme más conectado(a) con otras personas

- 1 (Nada importante) (1)
- 2 (2)
- 3 (3)
- 4 (4)
- 5 (Muy importante) (5)

Q66 Jeg tenkte det ville gi en distraksjon

- 1 (Ikke viktig i det hele tatt) (1)
- 2 (2)
- 3 (3)
- 4 (4)
- 5 (Veldig viktig) (5)

Q66 Ich dachte, dass es eine gute Ablenkung sein könnte

- 1 (Überhaupt nicht wichtig) (1)
- 2 (2)
- 3 (3)
- 4 (4)
- 5 (Sehr wichtig) (5)

Q66 Pensé que me distraería

- 1 (Nada importante) (1)
- 2 (2)
- 3 (3)
- 4 (4)
- 5 (Muy importante) (5)

Q67 Jeg tenkte det ville lette på humøret

- 1 (Ikke viktig i det hele tatt) (1)
- 2 (2)
- 3 (3)
- 4 (4)
- 5 (Veldig viktig) (5)

Q67 Ich dachte, dass es mich aufheitern könnte

- 1 (Überhaupt nicht wichtig) (1)
- 2 (2)
- 3 (3)
- 4 (4)
- 5 (Sehr wichtig) (5)

Q67 Pensé que me alegraría

- 1 (Nada importante) (1)
- 2 (2)
- 3 (3)
- 4 (4)
- 5 (Muy importante) (5)

Q68 Jeg ville oppdage ny musikk

- 1 (Ikke viktig i det hele tatt) (1)
- 2 (2)
- 3 (3)
- 4 (4)
- 5 (Veldig viktig) (5)

Q68 Ich neue Musik entdecken wollte

- 1 (Überhaupt nicht wichtig) (1)
- 2 (2)
- 3 (3)
- 4 (4)
- 5 (Sehr wichtig) (5)

Q68 Quería descubrir nueva música

- 1 (Nada importante) (1)
- 2 (2)
- 3 (3)
- 4 (4)
- 5 (Muy importante) (5)

Q121 Jeg kjenner artisten personlig

- 1 (Ikke viktig i det hele tatt) (1)
- 2 (2)
- 3 (3)
- 4 (4)
- 5 (Veldig viktig) (5)

Q121 Ich kenne den/die Künstler*in persönlich

- 1 (Überhaupt nicht wichtig) (1)
- 2 (2)
- 3 (3)
- 4 (4)
- 5 (Sehr wichtig) (5)

Q121 Conozco al artista personalmente

- 1 (Nada importante) (1)
- 2 (2)
- 3 (3)
- 4 (4)
- 5 (Muy importante) (5)

Q69 Andre grunner (vennligst beskriv)

________________________________________________________________

Q69 Andere Gründe (bitte beschreiben)

________________________________________________________________

Q69 Otras razones (Por favor descríbalas)

________________________________________________________________

Q70 I hvilken grad deltok du i interaksjoner gjennom konserten ved å lese, kommentere eller donere?

- 1 (Jeg leste ingen kommentarer) (1)
- 2 (2)
- 3 (3)
- 4 (4)
- 5 (Jeg leste mange kommentarer) (5)

Q70 Zu welchem Ausmaß haben Sie durch Lesen, Kommentieren, oder Spenden an Interaktionen im Rahmen des Konzerts teilgenommen?

- 1 (Ich habe keine Kommentare gelesen) (1)
- 2 (2)
- 3 (3)
- 4 (4)
- 5 (Ich habe viele Kommentare gelesen) (5)

Q70 ¿En qué medida participó en interacciones en torno al concierto leyendo, comentando o donando?

- 1 (No leí ningún comentario) (1)
- 2 (2)
- 3 (3)
- 4 (4)
- 5 (Leí muchos comentarios) (5)

Q71

- 1 (Jeg snakket ikke om konserten med noen) (1)
- 2 (2)
- 3 (3)
- 4 (4)
- 5 (Jeg snakket lenge om konserten, eller om konserten med mange folk) (5)

Q71

- 1 (Ich habe mit Niemandem über das Konzert gesprochen) (1)
- 2 (2)
- 3 (3)
- 4 (4)
- 5 (Ich habe ausführlich, bzw. mit mehreren Leuten über das Konzert gesprochen) (5)

Q71

- 1 (No hablé con nadie del concierto) (1)
- 2 (2)
- 3 (3)
- 4 (4)
- 5 (Hablé extensamente sobre el concierto con varias personas) (5)

Q72 Du kan velge flere alternativer nedenfor

- Jeg delte ikke konserten på nett (1)
- Jeg delte konserten på en sosial medie-plattform (2)
- Jeg delte konserten direkte med venner/familie (3)

Q72 Für die folgenden Antworten können Sie mehrere Optionen auswählen.

- Ich habe das Konzert nicht online geteilt (1)
- Ich habe das Konzert auf einer Social-Media-Plattform geteilt (2)
- Ich habe es direkt mit Familie oder Freunden geteilt (3)

Q72 Puede seleccionar varias opciones para las respuestas a continuación

- No compartí el concierto en línea (1)
- Compartí el concierto en una plataforma de redes sociales (2)
- Lo compartí directamente con familiares/amigo(a)s (3)

Q73 Du kan velge flere alternativer nedenfor

- Jeg kommenterte ikke i det hele tatt (1)
- Jeg klikket på et reaksjons-ikon (f.eks. lik eller hjerte (2)
- Jeg skrev en kort kommentar (3)
- Jeg skrev flere korte kommentarer (4)
- Jeg skrev en eller flere detaljerte kommentarer (5)

Q73 Für die folgenden Antworten können Sie mehrere Optionen auswählen.

- Ich habe überhaupt nicht kommentiert (1)
- Ich habe auf einen Reaktionsbutton geklickt (z.B. "Like") (2)
- Ich habe einen kurzen Kommentar geschrieben (3)
- Ich habe mehrere kurze Kommentare geschrieben (4)
- Ich habe einen oder mehrere detaillierte Kommentare geschrieben (5)

Q73 Puede seleccionar varias opciones para las respuestas a continuación

- No hice ningún comentario (1)
- Hice clic en un botón de reacción (Ej. Me gusta) (2)
- Escribí un comentario breve (3)
- Escribí varios comentarios breves (4)
- Escribí uno o más comentarios detallados (5)

Q119 Du kan velge flere alternativer nedenfor

- Artisten ba om donasjoner til dem selv (1)
- Artisten oppfordret til å donere til veldedighetsorganisasjoner (2)
- Artisten ba ikke om donasjoner (6)
- Annet (7) ________________________________________________

Q119 Für die folgenden Antworten können Sie mehrere Optionen auswählen.

- Der/die Künstler*in hat selbst um Spenden gebeten (1)
- Der/die Künstler*in hat zu Spenden an gemeinnützige Organisationen aufgerufen (2)
- Der/die Künstler*in hat nicht um Spenden gebeten (6)
- Andere (7) ________________________________________________

Q119 Puede seleccionar varias opciones para las respuestas a continuación

- El/la intérprete pidió donaciones por sí mismo(a) (1)
- El/la intérprete motivó a realizar donaciones para una organización (2)
- El/la intérprete no pidió ninguna donación (6)
- Otra (7) ________________________________________________

Q120 Du kan velge flere alternativer nedenfor

- Jeg donerte til artisten (1)
- Jeg donerte til en veldedighet som var anbefalt av artisten (2)
- Jeg kjøpte billett til konserten (3)
- Jeg donerte ikke (4)
- Andre måter du bidro på (5) ________________________________________________

Q120 Für die folgenden Antworten können Sie mehrere Optionen auswählen.

- Ich habe an den/die Künstler*in gespendet (1)
- Ich habe an eine gemeinnützige Organisation gespendet, die der/die Künstler*in empfohlen hat (2)
- Ich habe ein Ticket für die Veranstaltung gekauft (3)
- Ich habe nicht gespendet (4)
- Andere Art und Weise, auf die Sie unterstützt haben (5) ________________________________________________

Q120 Puede seleccionar varias opciones para las respuestas a continuación

- Hice una donación al/a la intérprete (1)
- Doné a una organización benéfica recomendada por el/la artista (2)
- Compré un boleto para el evento (3)
- No hice una donación (4)
- Otra forma en que contribuyó (5) ________________________________________________

Rate Vurder hvor enig du er i påstandene nedenfor.

Rate Bewerten Sie, inwieweit Sie den folgenden Aussagen zustimmen.

Rate Evalúe su nivel de acuerdo con las siguientes declaraciones.

Q74 Opplevelsen min under nettkonserten liknet på opplevelsene mine under en ekte konsert

- Helt uenig (1)
- Uenig (2)
- Verken uenig eller enig (3)
- Enig (4)
- Veldig enig (5)

Q74 Meine Erlebnisse mit dem Onlinekonzert sind ähnlich zu meinen Erlebnissen mit echten Konzerten.

- Stimme überhaupt nicht zu (1)
- Stimme eher nicht zu (2)
- Weder Zustimmung noch Ablehnung (3)
- Stimme eher zu (4)
- Stimme vollständig zu (5)

Q74 Mi experiencia en el concierto transmitido parecía similar a mis experiencias en un concierto real.

- Completamente en desacuerdo (1)
- En desacuerdo (2)
- No estoy ni en desacuerdo ni de acuerdo (3)
- De acuerdo (4)
- Muy de acuerdo (5)

Q75 Under nettkonserten hadde jeg en følelse av «å være der» med musikerne og publikummerne

- Helt uenig (1)
- Uenig (2)
- Verken uenig eller enig (3)
- Enig (4)
- Veldig enig (5)

Q75 Während ich das Onlinekonzert verfolgt habe, hatte ich den Eindruck des „Dort-seins“ mit den Künstler*innen und den anderen im Publikum.

- Stimme überhaupt nicht zu (1)
- Stimme eher nicht zu (2)
- Weder Zustimmung noch Ablehnung (3)
- Stimme eher zu (4)
- Stimme vollständig zu (5)

Q75 Mientras estaba en el concierto transmitido en vivo (streaming), tuve la sensación de "estar ahí" con lo(a)s artistas y los miembros de la audiencia.

- Completamente en desacuerdo (1)
- En desacuerdo (2)
- No estoy ni en desacuerdo ni de acuerdo (3)
- De acuerdo (4)
- Muy de acuerdo (5)

Q76 Jeg var helt fanget av nettkonserten

- Helt uenig (1)
- Uenig (2)
- Verken uenig eller enig (3)
- Enig (4)
- Veldig enig (5)

Q76 Von dem Onlinekonzert war ich völlig gefesselt.

- Stimme überhaupt nicht zu (1)
- Stimme eher nicht zu (2)
- Weder Zustimmung noch Ablehnung (3)
- Stimme eher zu (4)
- Stimme vollständig zu (5)

Q76 Estaba completamente cautivado(a) por el concierto transmitido en vivo (streaming).

- Completamente en desacuerdo (1)
- En desacuerdo (2)
- No estoy ni en desacuerdo ni de acuerdo (3)
- De acuerdo (4)
- Muy de acuerdo (5)

Q77 Jeg følte at jeg var til stede sammen med andre som var online under nettkonserten.

- Helt uenig (1)
- Uenig (2)
- Verken uenig eller enig (3)
- Enig (4)
- Veldig enig (5)

Q77 Ich hatte den Eindruck, in Gegenwart anderer Menschen zu sein, die ebenfalls online das Konzert verfolgt haben.

- Stimme überhaupt nicht zu (1)
- Stimme eher nicht zu (2)
- Weder Zustimmung noch Ablehnung (3)
- Stimme eher zu (4)
- Stimme vollständig zu (5)

Q77 Sentí que estaba en presencia de otras personas que estaban en línea en el concierto transmitido en vivo (streaming).

- Completamente en desacuerdo (1)
- En desacuerdo (2)
- No estoy ni en desacuerdo ni de acuerdo (3)
- De acuerdo (4)
- Muy de acuerdo (5)

Q78 Jeg følte at andre personer på nettkonserten var bevisst på min tilstedeværelse.

- Helt uenig (1)
- Uenig (2)
- Verken uenig eller enig (3)
- Enig (4)
- Veldig enig (5)

Q78 Ich hatte den Eindruck, dass die anderen Menschen im Onlinekonzert sich meiner Gegenwart bewusst waren.

- Stimme überhaupt nicht zu (1)
- Stimme eher nicht zu (2)
- Weder Zustimmung noch Ablehnung (3)
- Stimme eher zu (4)
- Stimme vollständig zu (5)

Q78 Sentí que las otras personas en el concierto transmitido en vivo (streaming) estaban al tanto de mi presencia.

- Completamente en desacuerdo (1)
- En desacuerdo (2)
- No estoy ni en desacuerdo ni de acuerdo (3)
- De acuerdo (4)
- Muy de acuerdo (5)

Q79 Personene på nettkonserten virket å være engasjerte og aktive med meg.

- Helt uenig (1)
- Uenig (2)
- Verken uenig eller enig (3)
- Enig (4)
- Veldig enig (5)

Q79 Die Menschen im Onlinekonzert erschienen mir gut eingebunden und aktiv zu sein.

- Stimme überhaupt nicht zu (1)
- Stimme eher nicht zu (2)
- Weder Zustimmung noch Ablehnung (3)
- Stimme eher zu (4)
- Stimme vollständig zu (5)

Q79 Las personas que estaban en el concierto en vivo (streaming) parecían estar atentos y activos hacia mí.

- Completamente en desacuerdo (1)
- En desacuerdo (2)
- No estoy ni en desacuerdo ni de acuerdo (3)
- De acuerdo (4)
- Muy de acuerdo (5)

Q80 Under nettkonserten var det stunder der datakommunikasjonen/grensesnittet virket å forsvinne, og det føltes ut som om jeg faktisk var der på konserten med publikum og musikerne.

- Helt uenig (1)
- Uenig (2)
- Verken uenig eller enig (3)
- Enig (4)
- Veldig enig (5)

Q80 Während des Onlinekonzerts gab es Momente, in denen das Computerinterface zu verschwinden schien und ich den Eindruck hatte, tatsächlich mit den Künstler*innen und dem Publikum in dem Konzert zu sein.

- Stimme überhaupt nicht zu (1)
- Stimme eher nicht zu (2)
- Weder Zustimmung noch Ablehnung (3)
- Stimme eher zu (4)
- Stimme vollständig zu (5)

Q80 Durante el concierto transmitido en vivo (streaming) hubo momentos en que la interfaz de la computadora pareció desaparecer, y sentí que realmente estaba en el concierto con el público y lo(a)s intérpretes.

- Completamente en desacuerdo (1)
- En desacuerdo (2)
- No estoy ni en desacuerdo ni de acuerdo (3)
- De acuerdo (4)
- Muy de acuerdo (5)

Q81 I følge din mening, var konserten bra?

- 1 (Ikke i det hele tatt) (1)
- 2 (2)
- 3 (3)
- 4 (4)
- 5 (Ekstremt bra) (5)

Q81 War das Konzert Ihrer Meinung nach gut?

- 1 (Überhaupt nicht) (1)
- 2 (2)
- 3 (3)
- 4 (4)
- 5 (Sehr) (5)

Q81 En su opinión, ¿el concierto fue bueno?

- 1 (Para nada) (1)
- 2 (2)
- 3 (3)
- 4 (4)
- 5 (Extremadamente) (5)

Q82 Hvor stort var publikummet (f.eks. 10, 100, 1000, eller usikker)?

________________________________________________________________

Q82 Wie groß war das Publikum?

________________________________________________________________

Q82 ¿Cuál era la cantidad de la audiencia? (Ej. 10, 100, 1000, o no estoy seguro(a))?

________________________________________________________________

Q83 Hvordan var kvaliteten på lyden?

- 1 (Veldig lav) (1)
- 2 (2)
- 3 (3)
- 4 (4)
- 5 (Perfekt) (5)

Q83 Wie war die Tonqualität?

- 1 (Sehr schlecht) (1)
- 2 (2)
- 3 (3)
- 4 (4)
- 5 (Sehr gut) (5)

Q83 ¿Cómo era la calidad del audio?

- 1 (Muy mala) (1)
- 2 (2)
- 3 (3)
- 4 (4)
- 5 (Excelente) (5)

Q84 Hvordan var kvaliteten på videoen?

- 1 (Veldig lav) (1)
- 2 (2)
- 3 (3)
- 4 (4)
- 5 (Perfekt) (5)

Q84 Wie war die Bildqualität?

- 1 (Sehr schlecht) (1)
- 2 (2)
- 3 (3)
- 4 (4)
- 5 (Sehr gut) (5)

Q84 ¿Cómo era la calidad del vídeo?

- 1 (Muy mala) (1)
- 2 (2)
- 3 (3)
- 4 (4)
- 5 (Excelente) (5)

End of Block: Block 2

Start of Block: Block 3

Q85 Praktiserer du musikk? (spiller instrument/synger, synger i kor, spiller i band etc.)

- Ja (1)
- Nei (2)

Q85 Machen Sie Musik (ein Instrument spielen/singen, in einem Chor singen, in einer Band spielen)?

- Ja (1)
- Nein (2)

Q85 ¿Tiene alguna experiencia en la práctica musical (tocar un instrumento / cantar, cantar en un coro, tocar en una banda)?

- Sí (1)
- No (2)

Q86 Hvor mange måneder musikktrening har du mottatt?

________________________________________________________________

Q86 Wie viele Monate musikalischer Ausbildung haben Sie erhalten?

________________________________________________________________

Q86 ¿Cuántos meses de instrucción musical ha recibido?

________________________________________________________________

Q87 Hvor mange timer i gjennomsnitt per dag bruker du på å lytte til musikk, enten mens du gjør noe annet eller som hovedaktivitet?

- 0 (1)
- 1-2 (2)
- 3-4 (3)
- 5-8 (4)
- 9 eller mer (5)

Q87 Wie viele Stunden täglich hören Sie durchschnittlich Musik, entweder während Sie etwas anderes tun oder als Ihre Hauptbeschäftigung?

- 0 (1)
- 1-2 (2)
- 3-4 (3)
- 5-8 (4)
- 9 oder mehr (5)

Q87 En promedio, ¿cuántas horas al día pasa realmente escuchando música, ya sea mientras hace otra cosa o como su actividad principal?

- 0 (1)
- 1-2 (2)
- 3-4 (3)
- 5-8 (4)
- 9 o más (5)

Q88 Hvor viktig har musikk vært for deg i løpet av de siste 3 årene av livet ditt?

- 1 (Ikke viktig i det hele tatt) (1)
- 2 (2)
- 3 (3)
- 4 (4)
- 5 (Ekstremt viktig) (5)

Q88 Wie wichtig war Musik in Ihrem Leben während der vergangenen 3 Jahre?

- 1 (Überhaupt nicht) (1)
- 2 (2)
- 3 (3)
- 4 (4)
- 5 (Sehr) (5)

Q88 ¿Qué tan importante ha sido la música en su vida los últimos 3 años?

- 1 (Nada) (1)
- 2 (2)
- 3 (3)
- 4 (4)
- 5 (Extremadamente) (5)

Q89 Hvilket oppmerksomhetsnivå har du vanligvis når du lytter til musikk?

- 1 (Kun bakgrunn) (1)
- 2 (2)
- 3 (3)
- 4 (4)
- 5 (Total konsentrasjon) (5)

Q89 Wie hoch ist normalerweise Ihre Aufmerksamkeit auf die Musik, während Sie Musik hören?

- 1 (Nur Hintergrundgeräusch) (1)
- 2 (2)
- 3 (3)
- 4 (4)
- 5 (Totale Konzentration) (5)

Q89 ¿Cuál es su nivel habitual de atención o de compromiso cuando escucha música?

- 1 (Solo de fondo) (1)
- 2 (2)
- 3 (3)
- 4 (4)
- 5 (Concentración total) (5)

Empathy Vennligst indiker i hvilken grad hver av de følgende påstandene beskriver deg:

Empathy Bitte geben Sie mithilfe der folgenden Skala an, zu welchem Ausmaß die Aussagen auf Sie zutreffen:

Empathy Indique en qué medida cada una de las siguientes afirmaciones lo describe a usted:

Q90 Når jeg ser noen bli utnyttet føler jeg for å beskytte dem på en eller annen måte.

- 1 (Beskriver meg ikke godt) (1)
- 2 (2)
- 3 (3)
- 4 (4)
- 5 (Beskriver meg veldig godt) (5)

Q90 Wenn ich beobachte, wie jemand ausgenutzt wird, fühle ich eine Art Beschützerinstinkt für diese Person.

- 1 (Beschreibt mich nicht gut) (1)
- 2 (2)
- 3 (3)
- 4 (4)
- 5 (Beschreibt mich sehr gut) (5)

Q90 Cuando veo que se aprovechan de alguien, siento algo de deseo de protegerlo(a).

- 1 (No me describe bien) (1)
- 2 (2)
- 3 (3)
- 4 (4)
- 5 (Me describe muy bien) (5)

Q91 Når jeg ser noen bli behandlet urettferdig hender det at jeg ikke syns særlig synd på dem.

- 1 (Beskriver meg ikke godt) (1)
- 2 (2)
- 3 (3)
- 4 (4)
- 5 (Beskriver meg veldig godt) (5)

Q91 Wenn ich beobachte, wie jemand unfair behandelt wird, empfinde ich manchmal kein besonders großes Mitleid für diese Person.

- 1 (Beschreibt mich nicht gut) (1)
- 2 (2)
- 3 (3)
- 4 (4)
- 5 (Beschreibt mich sehr gut) (5)

Q91 Cuando veo que alguien es tratado injustamente, a veces no siento mucha lástima por ello(a)s.

- 1 (No me describe bien) (1)
- 2 (2)
- 3 (3)
- 4 (4)
- 5 (Me describe muy bien) (5)

Q92 Jeg føler ofte ømhet og bekymring for mennesker som er mer vanskeligstilte enn meg.

- 1 (Beskriver meg ikke godt) (1)
- 2 (2)
- 3 (3)
- 4 (4)
- 5 (Beskriver meg veldig godt) (5)

Q92 Ich empfinde oft warmherzige Gefühle für Leute, denen es weniger gut geht als mir.

- 1 (Beschreibt mich nicht gut) (1)
- 2 (2)
- 3 (3)
- 4 (4)
- 5 (Beschreibt mich sehr gut) (5)

Q92 A menudo tengo sentimientos de ternura y preocupación por las personas menos afortunadas que yo.

- 1 (No me describe bien) (1)
- 2 (2)
- 3 (3)
- 4 (4)
- 5 (Me describe muy bien) (5)

Q93 Jeg vil beskrive meg selv som ganske bløthjertet.

- 1 (Beskriver meg ikke godt) (1)
- 2 (2)
- 3 (3)
- 4 (4)
- 5 (Beskriver meg veldig godt) (5)

Q93 Ich würde mich selbst als eine ziemlich weichherzige Person bezeichnen.

- 1 (Beschreibt mich nicht gut) (1)
- 2 (2)
- 3 (3)
- 4 (4)
- 5 (Beschreibt mich sehr gut) (5)

Q93 Me describiría a mí mismo como una persona de buen corazón

- 1 (No me describe bien) (1)
- 2 (2)
- 3 (3)
- 4 (4)
- 5 (Me describe muy bien) (5)

Q94 Noen ganger syns jeg ikke særlig synd på andre når de har problemer.

- 1 (Beskriver meg ikke godt) (1)
- 2 (2)
- 3 (3)
- 4 (4)
- 5 (Beskriver meg veldig godt) (5)

Q94 Manchmal tun mir Leute nicht leid, wenn sie Probleme haben.

- 1 (Beschreibt mich nicht gut) (1)
- 2 (2)
- 3 (3)
- 4 (4)
- 5 (Beschreibt mich sehr gut) (5)

Q94 A veces no siento tristeza por otras personas cuando tienen problemas.

- 1 (No me describe bien) (1)
- 2 (2)
- 3 (3)
- 4 (4)
- 5 (Me describe muy bien) (5)

Q95 Andre menneskers ulykke bryr meg som regel ikke så veldig.

- 1 (Beskriver meg ikke godt) (1)
- 2 (2)
- 3 (3)
- 4 (4)
- 5 (Beskriver meg veldig godt) (5)

Q95 Das Unglück anderer Menschen interessiert mich normalerweise nicht wirklich.

- 1 (Beschreibt mich nicht gut) (1)
- 2 (2)
- 3 (3)
- 4 (4)
- 5 (Beschreibt mich sehr gut) (5)

Q95 Generalmente no me afectan mucho las desgracias de otras personas

- 1 (No me describe bien) (1)
- 2 (2)
- 3 (3)
- 4 (4)
- 5 (Me describe muy bien) (5)

Q96 Jeg blir ofte ganske rørt av ting jeg ser.

- 1 (Beskriver meg ikke godt) (1)
- 2 (2)
- 3 (3)
- 4 (4)
- 5 (Beskriver meg veldig godt) (5)

Q96 Ich bin oft sehr berührt von den Dingen, die ich beobachte.

- 1 (Beschreibt mich nicht gut) (1)
- 2 (2)
- 3 (3)
- 4 (4)
- 5 (Beschreibt mich sehr gut) (5)

Q96 A menudo me conmueven las cosas que veo suceder.

- 1 (No me describe bien) (1)
- 2 (2)
- 3 (3)
- 4 (4)
- 5 (Me describe muy bien) (5)

Q118 Har du noen siste kommentarer til forskerne?

________________________________________________________________

Q118 Haben Sie noch Anmerkungen für die Versuchsleiter*innen?

________________________________________________________________

Q118 ¿Tiene algún comentario final para los investigadores?

________________________________________________________________

End of Block: Block 3

Start of Block: Link_to_raffle

Q122 Tusen takk for at du tok deg tid til å fullføre denne spørreundersøkelsen!

 Om du ønsker å delta i loddtrekningen av 20 gavekort fra Amazon eller Apple App Store & iTunes, kan du oppgi kontaktinformasjonen din (mailadresse) i et separat skjema: <https://nettskjema.no/a/148958>.

 Din kontaktinformasjon kan ikke bli koblet til svarene du har oppgitt i denne undersøkelsen, og mailadressen din vil ikke bli brukt til noe annet formål enn å kontakte deg om du skulle vinne et av gavekortene.

 Takk igjen for din innsats!

Q122 Vielen Dank, dass Sie sich Zeit genommen haben, an dieser Studie teilzunehmen!
  
Wenn Sie gerne an der Verlosung für insgesamt 20 Gutscheine für Amazon oder Apple App Store & iTunes teilnehmen möchten, können Sie Ihre Kontaktdaten (E-Mail-Adresse) in einer separaten Umfrage eintragen: <https://nettskjema.no/a/149015>
 
Ihre Kontaktdaten können nicht mit den Antworten in dieser Studie in Verbindung gebracht werden und Ihre E-Mail-Adresse wird ausschließlich dafür verwendet, Sie zu kontaktieren, falls Sie einen der Gutscheine gewonnen haben.
 
Nochmals vielen Dank für Ihre Mühe!

Q122 ¡Gracias por tomarse el tiempo para completar la encuesta!
Si desea participar en el sorteo de 20 vales de regalo para Amazon o Apple App Store e iTunes, puede dejar sus datos de contacto (dirección de correo electrónico) en una encuesta por separado: https://nettskjema.no/a/148928   Sus datos de contacto no se pueden vincular a sus respuestas en esta encuesta, y su dirección de correo electrónico no se utilizará para ningún otro propósito que no sea contactarlo(a) en caso de que gane uno de los vales de regalo.  ¡Una vez más gracias por su esfuerzo!

End of Block: Link_to_raffle

# S2: Pre-processing Details

The URL was used to determine the source or social media platform of the concert (e.g. YouTube, Facebook, other website, etc.). In cases where participants did not have the link, they were able to provide the name of the platform on which they watched the concert. Several participants reported using several social media platforms to view the concert, therefore only the first reported platform was used in analysis. There was only one sample that was viewed on “HouseParty”, a website where participants see other audience members, therefore it was re-coded to “Zoom” which has a similar viewing experience.

Participants reported the setting of the concert using a free text response. For analysis these were re-coded into settings of “Home” (included responses with words including “living”, “kitchen”, “desk”, and “office”, etc.), “Concert Hall” (included responses with words including “concert”, “hall”, “venue”, “church”, “stage”, etc.), and “Outdoors” (included responses with words including “park”, “field”, “garden”, “river”, etc.).

Participants’ reports of concert genre were manually re-coded into the STOMP-R genre classifications (Rentfrow & Gosling, 2003). Due to the Western bias of these genre classifications, instead of using “World” as a genre category, “Latin” and “Indian” were included to reflect the responses obtained from Chile and India.

After re-coding the genres, we found that there were several genres that had a small number of samples, therefore these genres were added to other genre categories. “Oldies” (n = 2), “Religious” (n = 3), “Soundtracks/theme song” (n= 2), and “Latin” (n = 3) were re-coded to “Pop”. “Punk” (n = 4) was re-coded to “Rock”. “Country” (n = 3) was re-coded to “Folk”. “New Age” (n = 2) was re-coded to “Dance/Electronica”. “Opera” was re-coded to Classical as was “Indian” (n = 2) because the music in this category was Indian Classical Music. “Soul/R&B/Funk” (n = 2) “Jazz” (n = 13) and “Rap/hip-hop” (n = 6) were all re-coded to a new genre category of “African-American genres” even though not all performers were African-American. Therefore the final genre classifications were African-American (n = 19), Classical (n = 43), Dance/electronica (n = 31), Folk (n = 43), Metal (n = 26), Opera (n = 8), Pop (n = 99), Rock (n = 84), and Various (n = 4).

Participants’ responses to the size of the audience were manually re-coded to provide a numeric estimate of audience size.

Relevant items from the Multimodal Presence Scale (MPS) were re-worded to increase their relevance to the virtual concert environment as recommended by the MPS authors (REF: Makransky et al., 2017). Specifically, items from the physical and social presence dimensions were used (see S1 for the questions). To evaluate if the structure of our adjusted presence scale was the same as the multimodal presence scale, we conducted a principal component analysis using the principal function from the psych toolbox (Revelle, 2020). It is theoretically likely that social presence and physical presence items would correlate, therefore we conducted an oblimin rotation to facilitate the interpretation of the component loadings. As expected, the principal component analysis revealed that there were two principal components with eigenvalues greater than 1 (component 1 = 3.477, component 2 = 1.216). We then proceeded by rotating the two retained components using oblimin rotation. The cumulative variance explained was 67% (component 1 = 36% and component 2 = 31%). We employed a cut-off threshold of +/-0.4 to determine which items loaded onto each factor. The items that loaded onto the first transformed component were #1. “My experience in the streamed concert seemed similar to my experiences in a real concert.” (0.75), #2. “While I was at the streamed concert, I had a sense of ‘being there’ with the performers and audience members.” (0.73), #3. "I was completely captivated by the streamed concert." (0.86), and #4. "During the streamed concert there were times where the computer interface seemed to disappear, and I felt like I was actually at the concert with the audience and performers" (0.69). The items that loaded onto the second transformed component were #5. "I felt like I was in the presence of other people who were online in the streamed concert." (0.70), #6. "I felt that the other people in the streamed concert were aware of my presence." (0.83), and #7. "The people in the streamed concert appeared to be engaged and active to me." (0.89). Based on the results of this analysis, the first component was interpreted as the physical presence subscale and the second component was interpreted as the social presence subscale. These subscales were averaged to provide separate measures of social and physical presence. These results are slightly different than the original MPS because item #4 originally loaded onto the social presence subscale but our re-wording made it load onto the physical presence subscale.

To assess the impact of the social distancing measures on the participants’ mental health, we collected measures of loneliness and anxiety. Specifically, participants were asked “Since the start of the social distancing measures, how often have you felt the following: i) loneliness, ii) lack of companionship, iii) isolated from others, and iv) anxiety. Response options were taken from the short scale for measuring loneliness in large surveys, but to maintain consistency with other items in the survey there were 5 response items: 1 (Hardly ever), 2, 3 (Some of the time), 4, 5 (Often) (REF: Hughes et al., short scale for measuring loneliness).

# S3: Supplementary Figures

**Figure S1**. The effect of the salience of the coronavirus during the concerts on (A) social connection and (B) *kama muta*


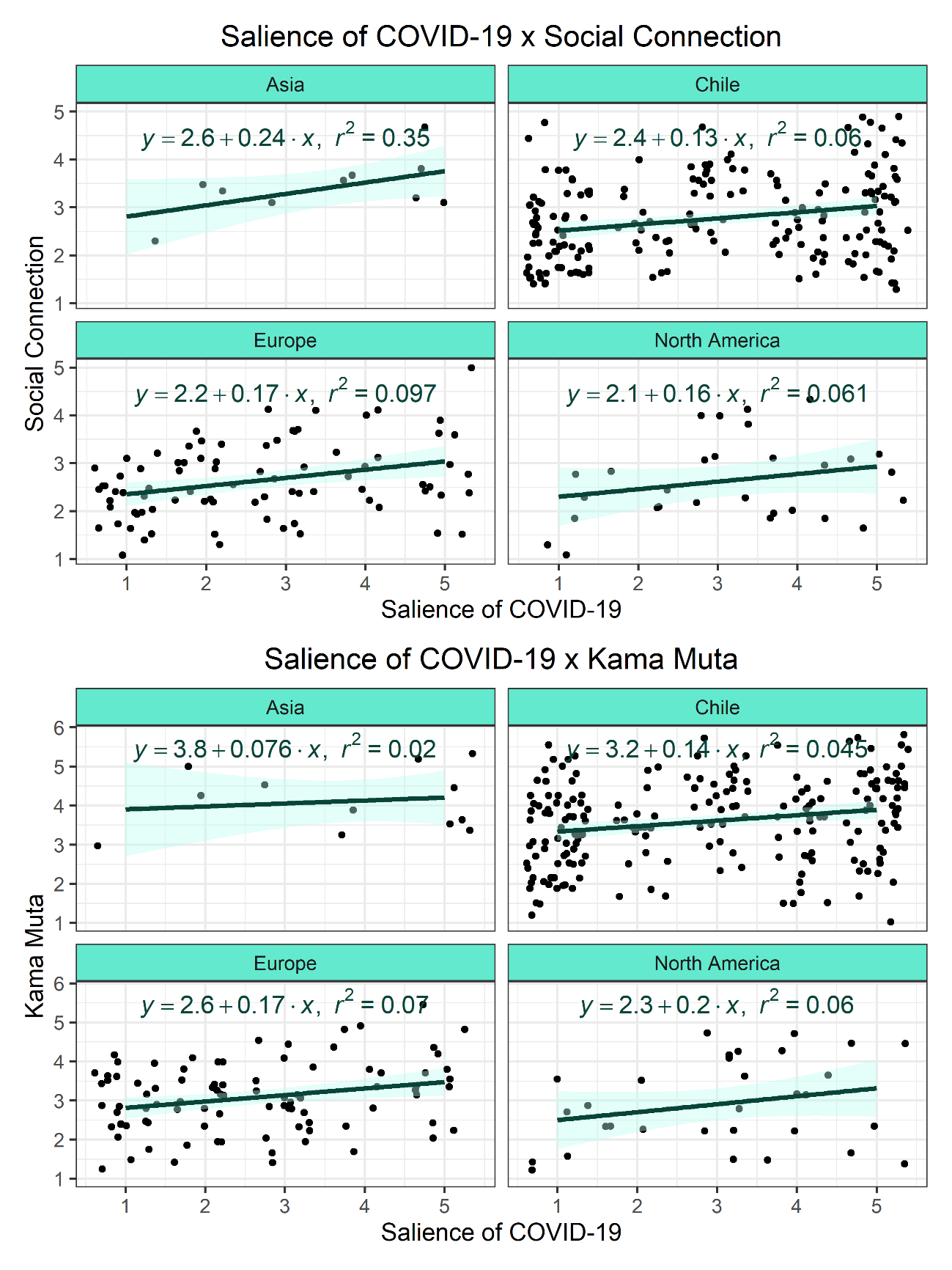


B

A

**Figure S2**. (A) The effect of genre on desire to move was significant such that all genres produced greater desire to move than classical music, (B) There was no significant effect of genre on relaxed breathing.


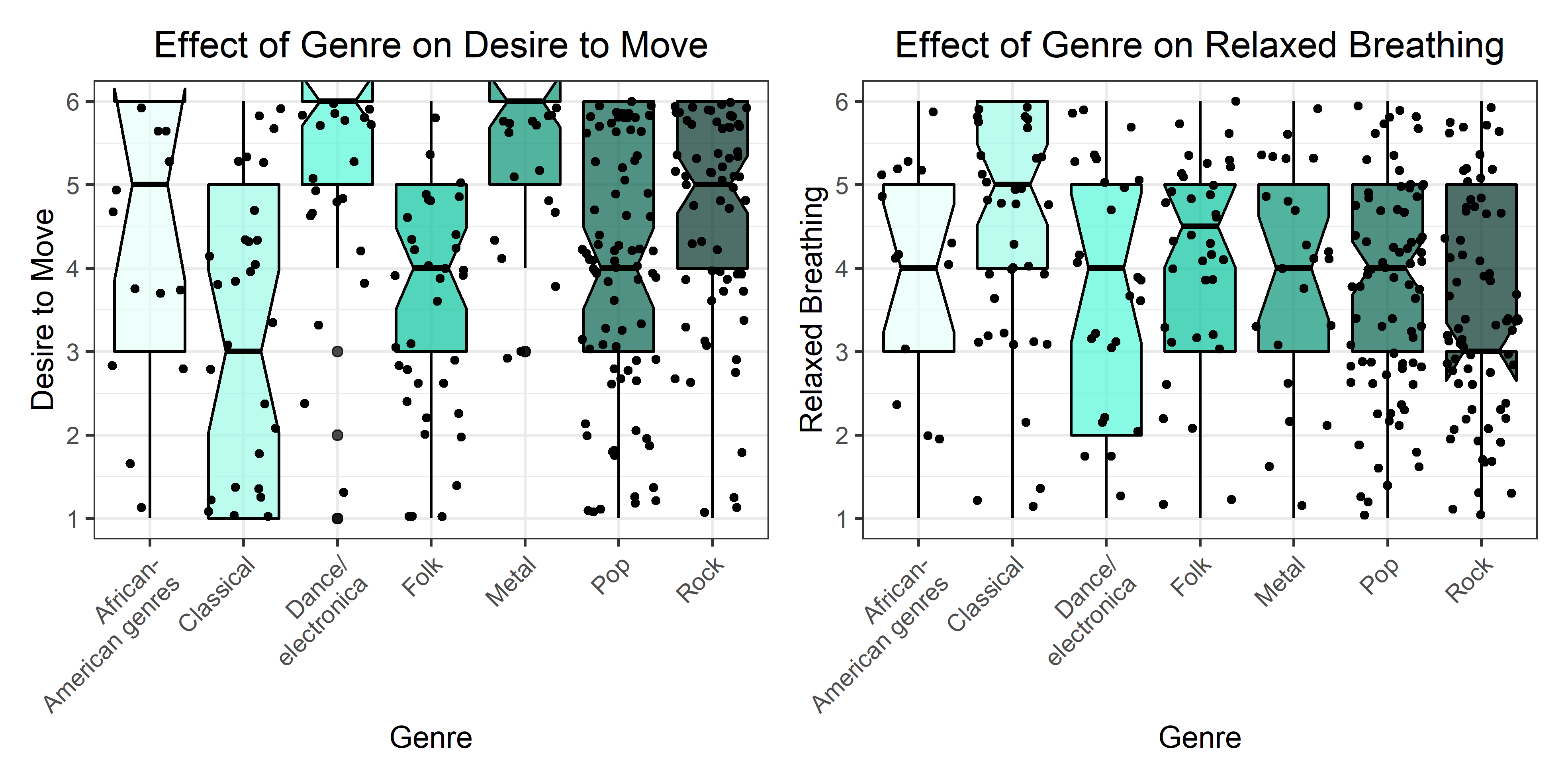


B

A

# Supplementary Tables

**Table S1**. The effect of musical genre on desire to move.

| **Predictors** | ***β*** | ***SE*** | ***p*** |  |
| --- | --- | --- | --- | --- |
| African-American Genres vs. Classical | 1.18 | 0.46 | 0.011 | * |
| Dance vs. Classical | 1.88 | 0.39 | 0 | *** |
| Folk vs. Classical | 0.74 | 0.35 | 0.034 | * |
| Metal vs. Classical | 2.27 | 0.4 | 0 | *** |
| Pop vs. Classical | 1.03 | 0.29 | 0.001 | *** |
| Rock vs. Classical | 1.76 | 0.3 | 0 | *** |

# Supplementary Data

Data and scripts for analysis can be found in the Open Science Foundation Quarantine Concerts: Publication repository (https://osf.io/skg7h/).
